# Profiling the specificity of clonally expanded plasma cells during chronic viral infection by single‐cell analysis

**DOI:** 10.1002/eji.202149331

**Published:** 2021-11-23

**Authors:** Daniel Neumeier, Alessandro Pedrioli, Alessandro Genovese, Ioana Sandu, Roy Ehling, Kai‐Lin Hong, Chrysa Papadopoulou, Andreas Agrafiotis, Raphael Kuhn, Danielle Shlesinger, Damiano Robbiani, Jiami Han, Laura Hauri, Lucia Csepregi, Victor Greiff, Doron Merkler, Sai T. Reddy, Annette Oxenius, Alexander Yermanos

**Affiliations:** ^1^ Department of Biosystems Science and Engineering ETH Zurich Basel Switzerland; ^2^ Institute of Microbiology ETH Zurich Zurich Switzerland; ^3^ Department of Immunology University of Oslo Oslo Norway; ^4^ Department of Pathology and Immunology University of Geneva Geneva Switzerland; ^5^ Division of Clinical Pathology Geneva University Hospital Geneva Switzerland

**Keywords:** viral infection, plasma cells, immune receptor repertoire sequencing, single‐cell, LCMV

## Abstract

Plasma cells and their secreted antibodies play a central role in the long‐term protection against chronic viral infection. However, due to experimental limitations, a comprehensive description of linked genotypic, phenotypic, and antibody repertoire features of plasma cells (gene expression, clonal frequency, virus specificity, and affinity) has been challenging to obtain. To address this, we performed single‐cell transcriptome and antibody repertoire sequencing of the murine BM plasma cell population following chronic lymphocytic choriomeningitis virus infection. Our single‐cell sequencing approach recovered full‐length and paired heavy‐ and light‐chain sequence information for thousands of plasma cells and enabled us to perform recombinant antibody expression and specificity screening. Antibody repertoire analysis revealed that, relative to protein immunization, chronic infection led to increased levels of clonal expansion, class‐switching, and somatic variants. Furthermore, antibodies from the highly expanded and class‐switched (IgG) plasma cells were found to be specific for multiple viral antigens and a subset of clones exhibited cross‐reactivity to nonviral and autoantigens. Integrating single‐cell transcriptome data with antibody specificity suggested that plasma cell transcriptional phenotype was correlated to viral antigen specificity. Our findings demonstrate that chronic viral infection can induce and sustain plasma cell clonal expansion, combined with significant somatic hypermutation, and can generate cross‐reactive antibodies.

## Graphical abstract

Single‐cell sequencing reveals clonally expanded plasma cells during chronic viral infection produce virus‐specific and cross‐reactive antibodies.

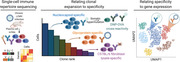

## Introduction

The humoral immune response plays a critical role in defending the host from a variety of pathogens, whereby the terminally differentiated plasma cell population contributes by secreting antibodies that can clear current and prevent future infection [1, 2]. Lymphocytic choriomeningitis virus (LCMV) represents a well‐studied pathogen that can establish chronic (dose‐dependent) infections in mice. Such infections can eventually be controlled and cleared due to the emergence of plasma cells secreting neutralizing antibodies. Circulating virus‐neutralizing antibodies are detected in the serum, but their plasma cell producers are mostly restricted to lymphoid tissues [3, 4]. It has further been established that neutralizing antibodies of the class‐switched IgG isotype are especially crucial for the resolution of chronic LCMV infection [5, 6]. These neutralizing IgG antibodies are directed against the LCMV surface glycoprotein complex (GPC) and only emerge several months after initial infection. In addition to neutralizing GPC‐targeting antibodies, GPC‐specific non‐neutralizing antibodies and antibodies targeting the nucleocapsid protein (NP) are also observed shortly after infection [5, 7]. Moreover, in addition to LCMV‐specific antibodies, it has been shown that chronic LCMV infection in mice also induces polyclonal hypergammaglobulinemia (PHGG), which induces the production of LCMV‐unspecific IgG antibodies. Induction of PHGG is mediated by LCMV‐specific nonfollicular CD4 helper cells, which support the activation of B cells in a BCR independent manner. For instance, LCMV infection results in plasma cells producing antibodies specific to unrelated antigens such as OVA and dinitrophenol (DNP) or autoantigens (i.e., ds and ss DNA, insulin, or thyroglobulin) [8, 9], whereby these autoantibody titers can even be comparable to the concomitantly induced antiviral antibody response [10].

The BM represents a stable niche for long‐lived plasma cells (BM PCs) [11]. In this terminal differentiation state, BM PCs have strongly reduced surface antibody (BCR) expression in exchange for an increased antibody secretion capacity [12, 13, 14]. This decreased, yet nevertheless present (Blanc et al., 2016; Pinto et al., 2013), antibody surface expression complicates the identification and subsequent isolation of virus‐specific and class‐switched plasma cells by means of traditionalFACS methods. It has previously been shown that LCMV‐specific plasma cells reside in the BM following infection [13] via ELISpot assays [15, 16], in which antigen specificity for a given target can be quantified. However, such assays do not allow for recovery of the underlying antibody sequences and, therefore, information regarding the relationship between clonal expansion, somatic hypermutation, and affinity to viral antigens cannot be obtained. Furthermore, these assays do not allow a detailed transcriptional characterization of individual antibody‐secreting cells.

Recent advances in single‐cell sequencing (scSeq) technologies have made it possible to obtain both transcriptome and antibody repertoire information from single B cells at a large scale [17, 18, 19, 20] enabling an integrated analysis of genotypic and phenotypic metrics of paired antibody repertoires.

Here, we performed scSeq to molecularly quantify both the antibody repertoire and transcriptome of thousands of murine BM PCs from a mouse following chronic viral infection and compare this with mice immunized with protein antigens. Importantly, we also took advantage of scSeq data to reconstruct full‐length antibody proteins from BM PCs to screen for antigen specificity. Such extensive scSeq combined with experimental antibody characterization (synthetic gene construction, cloning, recombinant expression, and antigen‐binding assays) limited our capacity to perform this on only a single chronically infected LCMV mouse. Nevertheless, the single‐cell linking of molecular genotype, phenotype, and antigen specificity of B cells has rarely been achieved before. One of the only examples to date has been in the context of human influenza vaccination and was also confined to a single individual [18]. We report that relative to protein immunization, chronic viral infection resulted in an increased proportion of clonally expanded, class‐switched, and somatically hypermutated BM PCs. We could additionally demonstrate that highly expanded IgG‐secreting BM plasma cells produce virus‐specific, cross‐reactive, and potentially autoreactive antibodies. Finally, single‐cell transcriptome sequencing analysis suggests that plasma cells producing antibodies specific for the LCMV GPC protein occupy distinct transcriptional states relative to plasma cells secreting NP‐specific antibodies. Together, our findings inform the relationship between clonal expansion, somatic hypermutation, gene expression, and antigen specificity during chronic LCMV infection.

## Results

### Chronic viral infection results in high clonal expansion of plasma cells expressing class‐switched IgG

**Figure 1:**
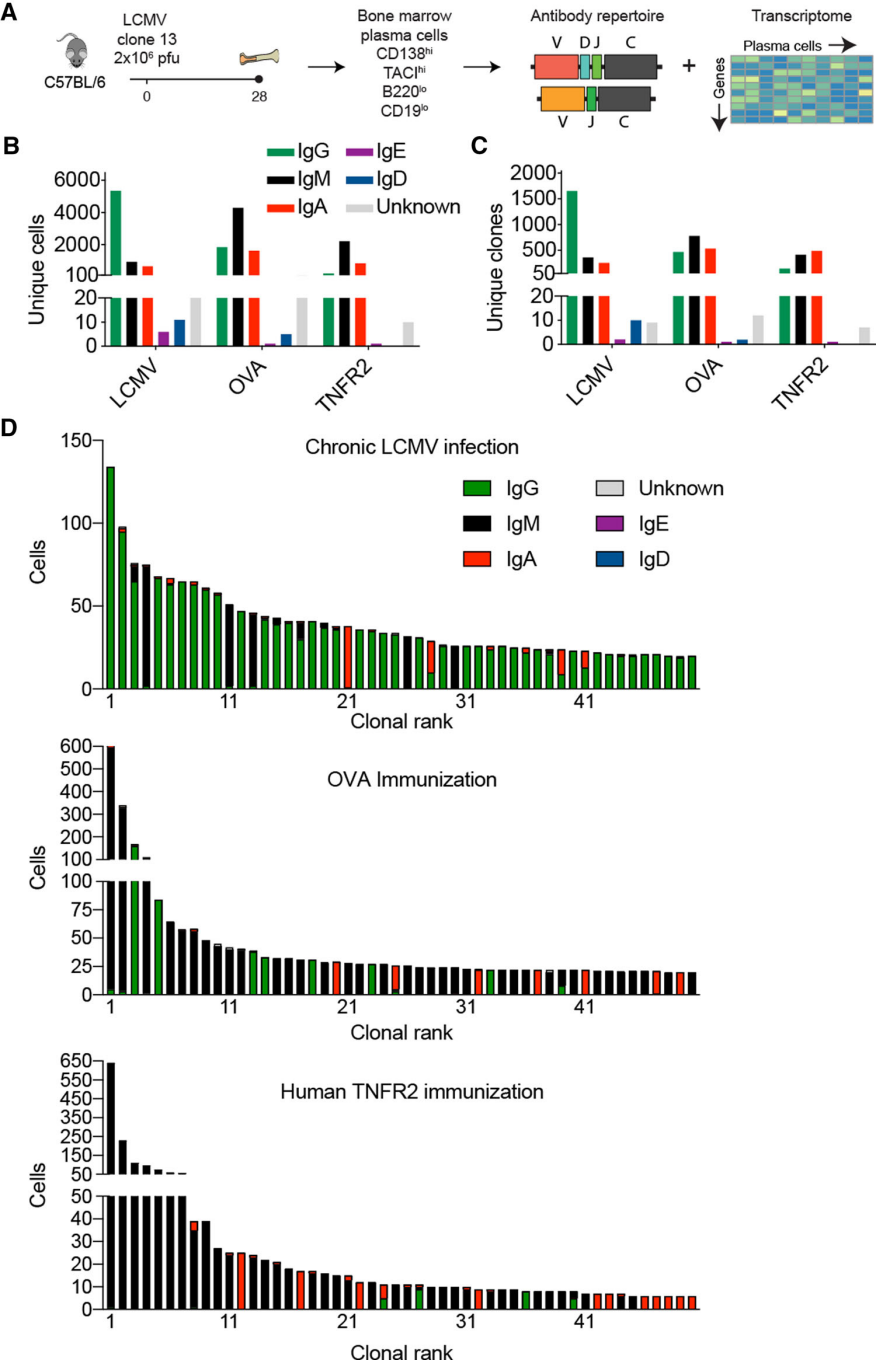
Single‐cell immune repertoire sequencing reveals clonal expansion and class‐switching in the BM plasma cell repertoire following chronic viral infection. (A) Experimental overview of chronic LCMV infection, BM plasma cell isolation, and single‐cell sequencing. (B) Number of cells per isotype for each infected or immunized mouse. Only cells containing exactly one variable heavy (V_H_) and variable light (V_L_) chain were considered. Colors correspond to isotype. (C) Number of clones per isotype for each infected or immunized mouse. Clones were determined by grouping those B cells containing identical CDRH3 + CDRL3 AA sequences. The isotype was determined as the isotype corresponding to the majority of cells within one clone. Color corresponds to isotype. (D) Clonal expansion for the top 50 most expanded clones of the BM plasma cells for each infected or immunized mouse. Clones were determined by grouping those B cells containing identical CDRH3 + CDRL3 AA sequences. Color corresponds to isotype.

To molecularly characterize the antibody repertoire and gene expression profiles following chronic viral infection, we isolated BM PCs 28 days postinfection (dpi) with high‐dose (2 × 10^6^ focus forming units [ffu]) LCMV clone 13 (Fig. 1A). We performed FACS to isolate BM PCs based on CD138+, TACI+ CD19^lo^, and B220^lo^ expression (as previously described) [21] and performed scSeq of transcriptomes and antibody repertoires using the 5’ GEX and V(D)J protocols from 10× Genomics. Additionally, we performed scSeq of BM PC from mice that had undergone serial immunizations with the protein antigens OVA or the extracellular domain of human TNF receptor 2 (TNFR2; 61% AA sequence homology to its murine homolog); this allowed us to compare transcriptome and repertoire features of chronic infection to protein immunization (Supporting information Fig. S1A and S1B). We were able to recover thousands of cells for each mouse (Supporting information Fig. S1C and S1D), with chronic LCMV infection resulting in the highest proportion of class‐switched IgG producing BM PCs and clones (Fig. 1B and C, Supporting information Fig. S1E and S1F): we detected 5337 IgG‐expressing BM PCs (Fig. 1B) corresponding to 1653 unique clones (Fig. 1C). Unique clones are defined herein as cells expressing only one heavy and one light chain and containing identical CDR 3 variable heavy and CDR 3 variable light chain (CDRH3 + CDRL3) AA sequences. The discrepancy between the number of cells and the number of unique clones for all mice and isotypes suggested significant clonal expansion in the BM PC repertoires. Upon closer inspection of the 50 most expanded clones in each mouse (Fig. 1D), we observed a striking difference in the proportion of IgG expressing BM PCs: 46 clones were expressing IgG, with 10 clones showing high clonal expansion (composed of more than 50 distinct cells) in chronic virus‐infected mice compared to only four IgG expressing clones, with less clonal expansion in both protein immunized mice (Fig. 1D).

**Figure 2:**
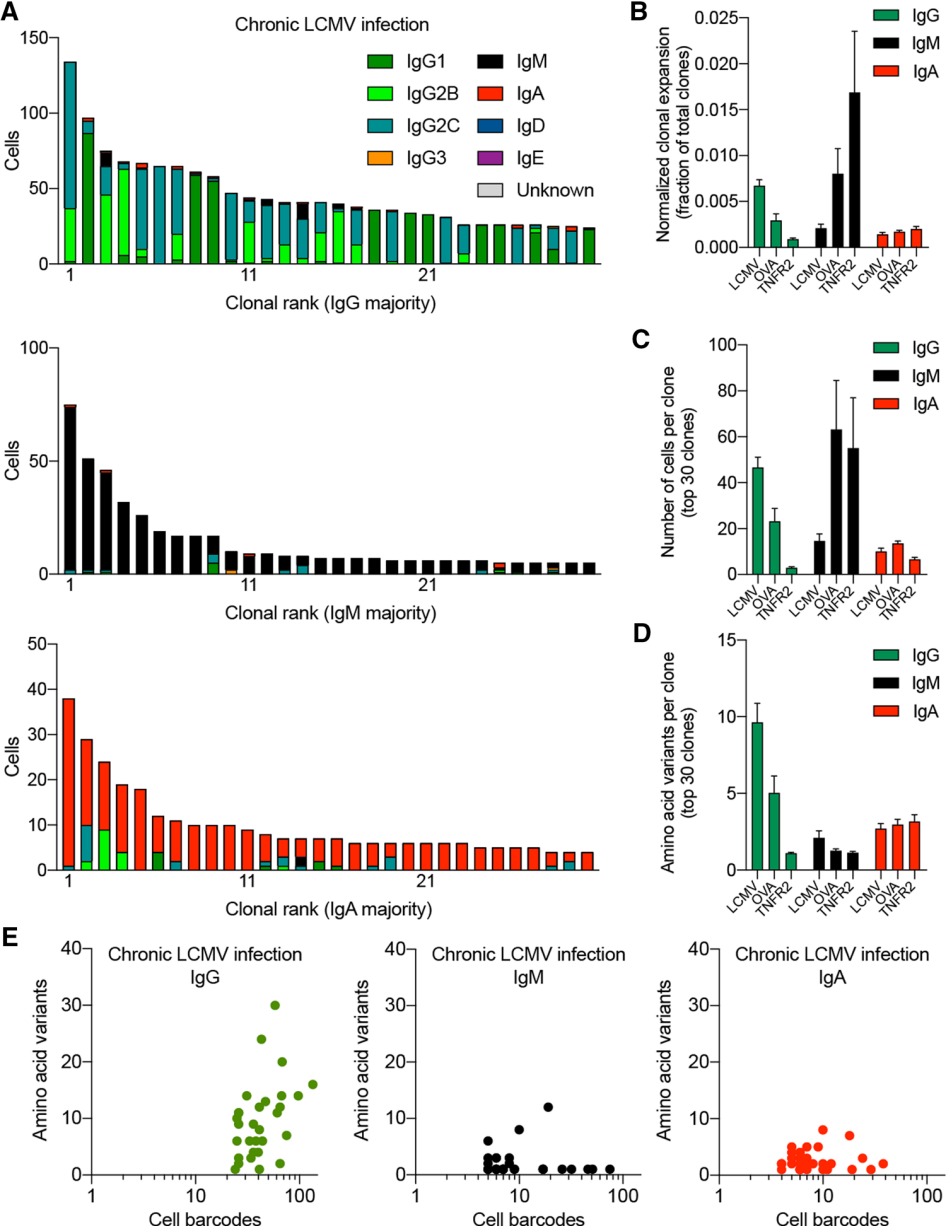
Clonal expansion and number of antibody variants in the IgG BM plasma cell repertoire following chronic viral infection as compared to protein immunizations. (A) Clonal expansion of the BM plasma cells (PC) separated by isotype for a single mouse following chronic LCMV infection. The number of distinct cell barcodes belonging to the top 30 clones is shown. Clone was determined by grouping those B cells containing identical CDRH3 + CDRL3 AA sequences. Only cells containing exactly one variable heavy (V_H_) and variable light (V_L_) chain AA sequence were considered. The isotype was determined as the isotype corresponding to the majority of cells within one clone. Color corresponds to isotype. (B) Normalized clonal expansion separated by isotype for each of the infected or immunized mice. The number of cell barcodes per clone was divided by the total number of cells in each repertoire. (C) Number of cells per clone for the top 30 clones separated by isotype for each of the infected or immunized mice. (D) Quantity of AA variants within each clone separated by isotype for each of the infected or immunized mice. AA variants were determined by quantifying the number of unique full‐length V_H_ + V_L_ AA sequence variants within each clone. Clone was determined by grouping those B cells containing identical CDRH3 + CDRL3 AA sequences. (E) Relationship between the number of unique AA variants and the number of cell barcodes for the top 30 clones separated by isotype majority following chronic LCMV infection.

Given that the majority of clonally expanded BM PCs expressed IgG during chronic LCMV infection, we next determined the fraction that expressed the IgG2c subtype, as IgG2c is the most dominant isotype of LCMV‐specific antibodies and has previously been shown to be crucial for viral neutralization [22]. Further analysis of the expanded IgG clones revealed heterogeneous expression of IgG1, IgG2b, and IgG2c following chronic viral infection, but not following protein immunizations (Fig. 2A, Supporting information Fig. S2A and S2B). Interestingly, in the case of chronic viral infection, clones predominantly expressing IgG1 did not contain many cells producing the other IgG subtypes, whereas clones predominantly expressing IgG2b and IgG2c contained more variation in subtypes. Quantifying the clonal expansion across the three BM PC repertoires suggested that chronic viral infection increased both the proportion and number of clonally expanded cells expressing IgG, coinciding with a decrease in the proportion and number of clonally expanded IgM cells relative to protein immunizations (Fig. 2B and C).

### Chronic viral infection drives high levels of somatic hypermutation in clonally expanded plasma cells

After observing that the majority of recovered plasma cells were of the IgG isotype, we hypothesized the most expanded IgG clonal families would contain more somatic hypermutation variants than highly expanded IgM and IgA clones following both viral infection and immunization. We quantified the number of somatic variants (defined by unique, full‐length AA sequence of paired V_H_ and V_L_) for the 30 most expanded clones of each isotype. Our analysis revealed an increased number of somatic variants of the IgG isotype compared to clones of the IgA or IgM isotype for both LCMV infection and OVA immunization, but not for immunization with TNFR2 (Fig. 2D, Supporting information Fig. S3). Relating the number of cells to the number of unique antibody variants demonstrated an increased Pearson correlation (*r* = 0.39) for the IgG isotype relative to IgM (*r* = 0.09) and IgA (*r* = 0.04), but nevertheless indicated that the most expanded clones are not necessarily those containing the largest sequence variation (Fig. 2E). While this increase in the number of contemporary clonally related antibody variants may not be directly induced by infection or immunization, it is nevertheless interesting that certain highly expanded IgM clones (>100 cells) populate the PC repertoire and produce entirely identical antibodies.

### Chronic viral infection results in plasma cells with inflammatory and isotype‐specific gene expression signatures

**Figure 3:**
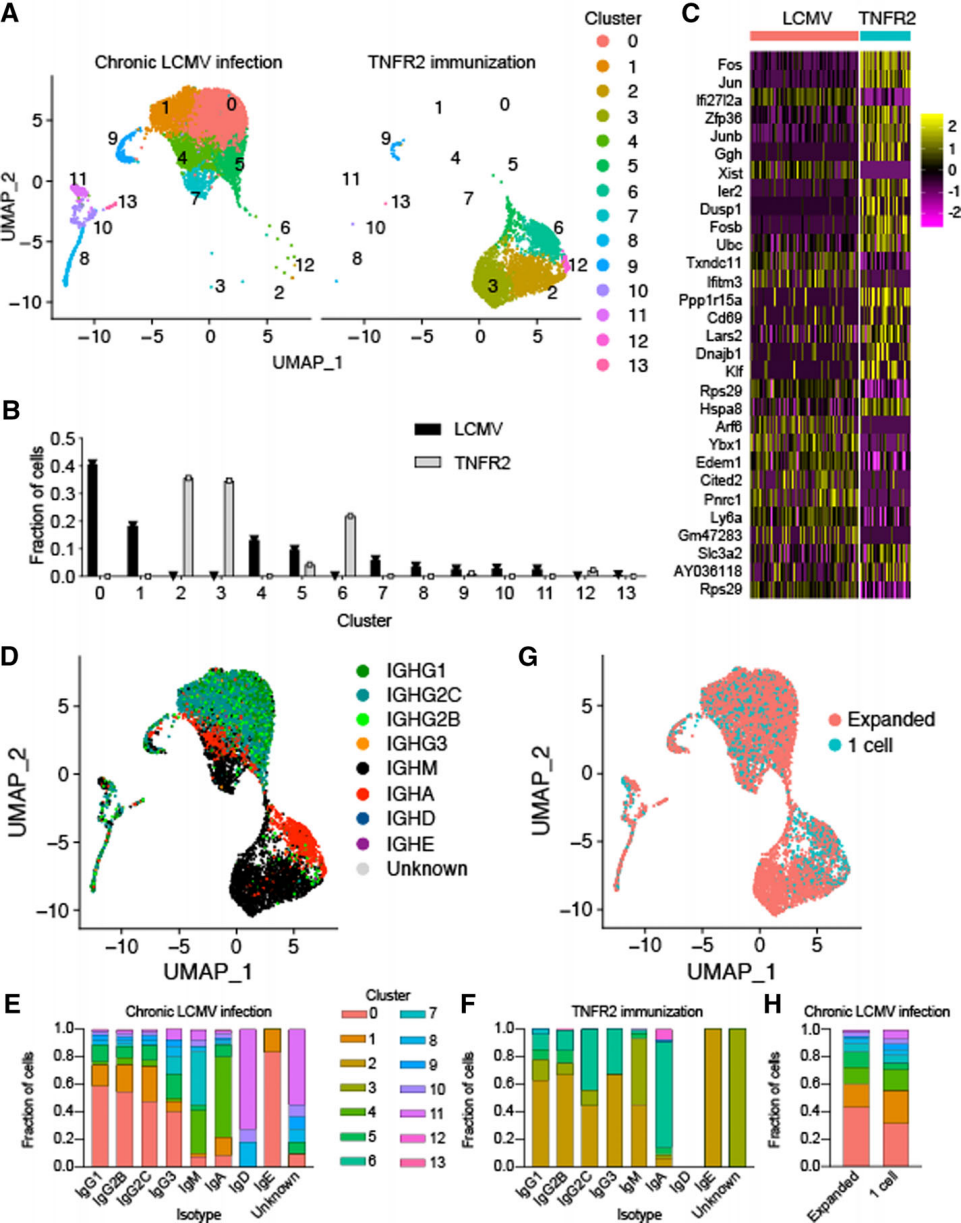
Transcriptional heterogeneity of BM PCs from LCMV‐infected or TNFR2‐immunized mice. (A) Uniform manifold approximation projection (UMAP)‐based total gene expression for BM plasma cell (BM PC) repertoire following either LCMV infection or TNFR2 immunization. Each point corresponds to a cell and color corresponds to the transcriptional cluster. (B) The fraction of cells belonging to each transcriptional cluster from the BM PC repertoire following either LCMV infection or TNFR2 immunization. (C) Differentially expressed genes between BM PCs following LCMV infection and TNFR2 immunization. The order of genes (from top to bottom) corresponds to the highest average log‐fold change. All genes displayed have adjusted *p* values < 0.01. (D) UMAP displaying isotype distribution of BM PCs following either LCMV infection or TNFR2 immunization. Each point corresponds to a cell and color indicates isotype based on VDJ sequencing data. € Cluster membership separated by isotype for the BM PCs following LCMV infection. Color indicates the transcriptional cluster from panel A. (F) Cluster membership separated by isotype for the BM PCs following TNFR2 immunization. Color indicates the transcriptional cluster from panel A. (G) UMAP displaying clonal expansion for the BM PCs following either LCMV infection or TNFR2 immunization. “Expanded” corresponds to those clones supported by two or more unique cell barcodes. Clone was determined by grouping those B cells containing identical CDRH3 + CDRL3 AA sequences. (H) Cluster membership separated by clonal expansion for the BM PCs following either LCMV infection or TNFR2 immunization. Color indicates the transcriptional cluster from panel A.

We next leveraged the ability to simultaneously perform the whole transcriptome‐ as well as targeted V_H_/V_L_‐repertoire scSeq to determine unique gene expression profiles of PC clones from chronic LCMV infection or TNFR2 immunization (scSeq of transcriptomes for OVA immunized mouse was not performed). We first filtered out all cells not found in both the scSeq antibody repertoire and transcriptome datasets for the virus‐infected and immunized mice, which resulted in 6446 and 3069 cells, respectively (Fig. 3A). We next removed those genes related to the adaptive immune receptor (e.g., IgHV1‐1, IgHM) to reduce the potential influence that clonal relationships could exert on transcriptional profiles. After normalizing and scaling gene expression counts for the two sequencing libraries, we performed unsupervised clustering to group cells with similar transcriptional profiles in an unbiased manner and visualized each cell in two dimensions using uniform manifold approximation projection (UMAP), which resulted in 14 clusters with distinct transcriptional signatures (Fig. 3A, Supporting information Fig. S4, Table S1). Quantifying the cluster membership for each sample confirmed the visual observation from the UMAP that the BM PCs from viral infection occupy almost entirely different clusters than the BM PCs following protein immunization, with minor overlap occurring only in clusters 5 and 9 (Fig. 3B). We next computed the differentially expressed genes between the samples, which revealed an upregulation in IFN‐related genes (*Ifi27l2a* and *Ifitm3*) and a downregulation of genes involved in Fos and Jun signaling pathways (*Fos*, *Fosb*, *Jun*, *Junb*, *Ubc*) in BM PCs from chronic LCMV infection (Fig. 3C, Supporting information Table S2). While it is likely that batch effects play a role in these differences, expression of traditional plasma‐cell markers (*Sdc1* encoding CD138, *Tnfrsf13b* encoding TACI, *Slamf7*, *Prdm1* encoding *BLIMP1*) were nevertheless comparable between the two samples (Supporting information Fig. S5A and 5B).

Since, we initially observed that TNFR2 immunization and LCMV infection resulted in plasma cells with divergent class‐switching profiles (Fig. 1D, Supporting information Fig. S1E and S1F), we next questioned whether the sample‐specific clustering could be partially explained by isotype‐specific gene expression signatures. We, therefore, overlaid isotype information for each cell based on the antibody repertoire scSeq data, which separated the cells into IgG‐, IgM‐, and IgA‐expressing plasma cells (Fig. 3D). Quantifying the cluster membership for all cells of a given isotype demonstrated comparable distributions across the IgG subtypes, which diverged from cells expressing either IgA (cluster 4) or IgM (clusters 4 and 7) (Fig. 3E), with exclusive genes defining IgG (*Ly6c2*, *Slpi*), IgA (*Ccl10*, *Glpr1*), and IgM (*Ggh*, *Ptpn6*) (Supporting information Fig. [Link], [Link], [Link], [Link]). A similar isotype‐specific clustering effect was also observed following TNFR2 immunization (Fig. 3F), suggesting this effect was not specific to viral infection. Performing an identical analysis using only the PCs from chronic LCMV infection demonstrated similar isotype‐specific clustering (Supporting information Fig. S7A and S7B). As we removed Ig genes before gene expression analysis, we questioned whether the observed differences in isotype may be explained by different fractions of non‐Ig genes. Quantifying all cells from both LCMV‐infected and TNFR2‐immunized mice demonstrated that, on average, 42 and 30% of total counts per cell were not related to the immune receptor for PCs from chronic LCMV and TNFR2‐immunization, respectively (Supporting information Fig. S7C). Analyzing the major isotypes separately revealed that Ig‐filtering removed similar number of counts per cell for all isotypes (Supporting information Fig. S7D). Although quantifying the most‐abundant isotypes separately revealed a trend that IgM‐producing PCs contained less immune receptor‐related transcript than other isotypes, there were minor differences between isotypes for both the TNFR2‐immunized and LCMV‐infected mice (Supporting information Fig. S7D).

We next asked whether expanded PC clones (clones supported by more than one cell) were transcriptionally distinct compared to unexpanded clones (clones supported by only one single cell). Visualizing and quantifying the cluster membership of the expanded and unexpanded clones following chronic LCMV infection demonstrated similar transcriptional profiles between the two groups (Fig. 3G and H), implying that either clonal expansion does not result in persisting transcriptional programs in BM PCs or that we have not sampled enough cells to sufficiently label unexpanded cells.

### Clonally expanded plasma cells express non‐neutralizing antibodies with specificity to viral antigens

**Figure 4:**
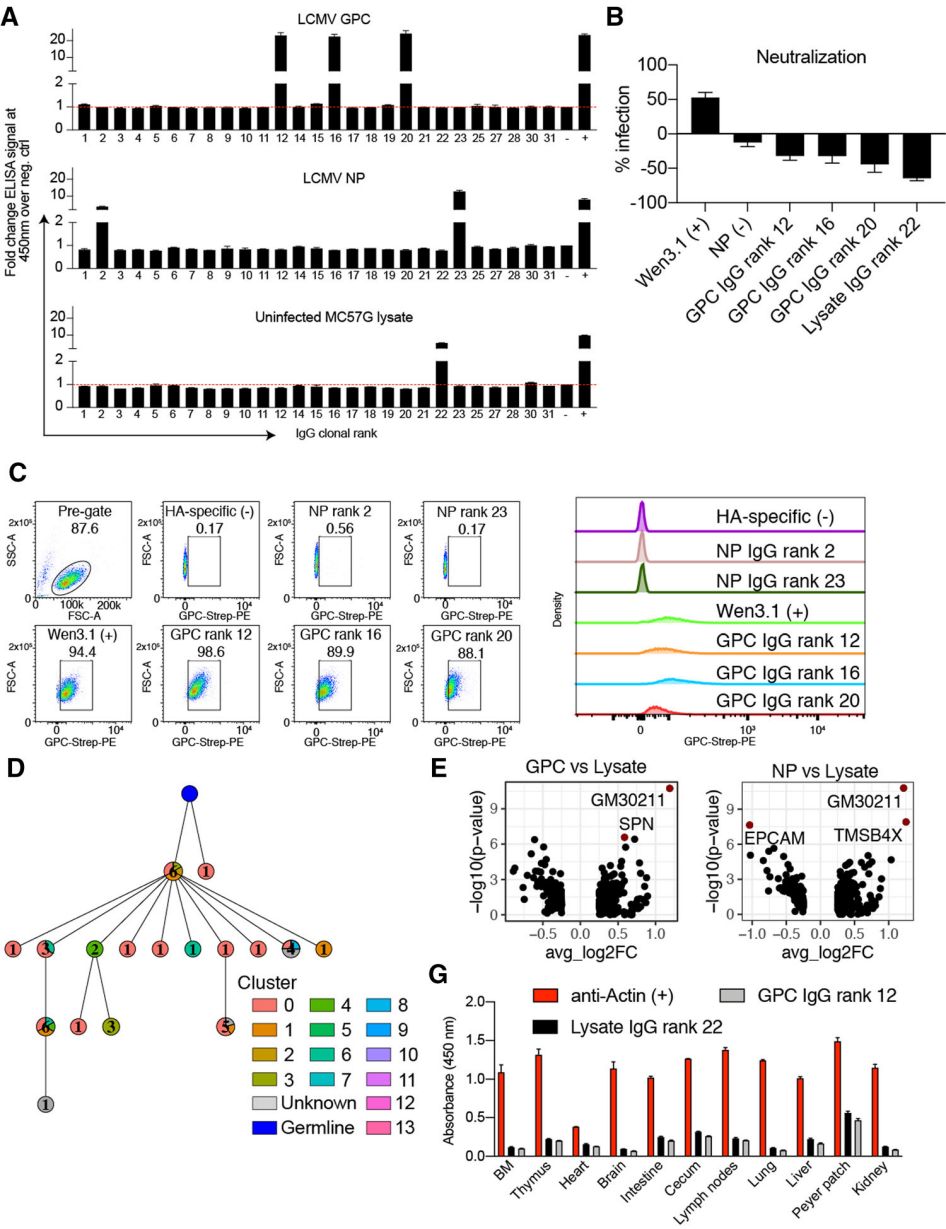
Clonally expanded plasma cells are virus‐specific and potentially autoreactive. (A) The ELISA signal of duplicate measurements at 450 nm is shown relative to a negative background control (red dotted line indicates background level). Clone was determined by grouping those B cells containing identical CDRH3 + CDRL3 AA sequences. Only cells containing exactly one variable heavy (V_H_) and variable light (V_L_) chain were considered. The isotype was determined as the isotype corresponding to the majority of cells within one clone. For each clone, the antibody variant (combined V_H_+V_L_ nucleotide sequence) supported by the most unique cell barcodes was selected to be expressed. Clones 13, 24, 26, and 29 did not express in our hybridoma system and were, therefore, excluded from the ELISA. (B) Neutralization potential of the GPC‐ and lysate‐binding clones normalized by wells without antibody. (C) Flow cytometry confirmed GPC‐binding inferred by ELISA of antibody expressing hybridomas. (D) Mutational network of the IgG clone binding MC57G lysate. Nodes represent unique antibody variants (combined V_H_ + V_L_ nucleotide sequence) and edges demonstrate sequences with the smallest separation calculated by edit distance. Node color corresponds to transcriptional cluster from 3A. The size and label of the nodes indicate how many cells express each full‐length antibody variant. Clone was determined by grouping those B cells containing identical CDRH3 + CDRL3 AA sequences. Only cells containing exactly one variable heavy (V_H_) and variable light (V_L_) chain were considered. The isotype was determined as the isotype corresponding to the majority of cells within one clone. The germline node represents the unmutated reference sequence determined by 10× Genomics cell ranger. (E) Differentially expressed genes between LCMV NP, GPC binders, and MC57G lysate binders. Those labeled and colored points correspond to those genes with an adjusted *p* value of < 0.01. G. ELISA on lysate from various tissues of naive C57BL/6 mice. Error bars indicate the SE of mean.

After observing both high levels of clonal expansion and somatic variants of the IgG isotype in plasma cells of LCMV‐infected mice, we next evaluated whether this correlated to antibodies with binding specificity to LCMV antigens. To this end, the scSeq antibody repertoire allowed us to directly synthesize paired V_L_‐C_k_‐2A‐V_H_ cassettes which were cloned into a custom vector for the generation of stable hybridoma cell lines using CRISPR/Cas9 [23, 24]; this way, we recombinantly expressed and validated the specificity for the 31 most expanded IgG clones from the BM PC repertoire (Supporting information Table S6). We first investigated the specificity of each of the antibodies by ELISA by testing for binding to the following antigens: purified recombinant NP or GPC from LCMV clone 13, lysate from uninfected and LCMV‐infected MC57G cells (a C57BL/6‐derived fibroblast cell line; Battegay et al., 1991), insulin, dsDNA, and DNP‐OVA. Two of the IgG clones displayed clear reactivity against the viral NP protein, and three against GPC, all five of which showed binding to the lysate of infected cells (Fig. 4A, Supporting information Fig. S8). Surprisingly, one clone demonstrated reactivity to both the uninfected and infected MC57G lysate (Fig. 4A, Supporting information Fig. S8), potentially indicating the presence of a clonally expanded BM PC producing autoreactive antibodies. Transiently expressing the GPC‐ and lysate‐specific antibodies and performing a neutralization assay [13] demonstrated that these antibodies could not prevent in vitro infection of MC57G cells relative to a known neutralizing monoclonal antibody (Fig. 4B). Although this is somewhat expected as serum neutralizing antibodies are often not detectable before approximately 40 dpi ([13] (Greczmiel et al., 2021), we nevertheless wanted to confirm that these antibodies were indeed GPC‐specific. To this end, we leveraged the antibody surface presentation of our hybridoma cell lines to perform flow cytometry using recombinantly produced LCMV GPC‐strep. Staining with an antistreptag antibody confirmed that the three clones, in addition to the positive control, were indeed GPC‐reactive (Fig. 4C). This further demonstrated that the two NP‐specific and lysate‐specific clones did not exhibit GPC reactivity (Fig. 4C).

### Lysate‐reactive clone contains somatic variants, non‐distinct transcriptional signatures, and does not bind uninfected tissue lysates

Given the unexpected observation that an expanded IgG clone displayed reactivity to naïve B6 lysate, we questioned whether any repertoire or transcriptional features would indicate autoreactivity. We first assigned BM PCs to clonal lineages by grouping cells using identical V and J germline genes for both V_H_ and V_L_ chains, identical CDRH3 and CDRL3 sequence length, and sharing 70% AA sequence homology across CDRH3 + CDRL3. We then calculated mutational networks by computing distance matrices based on the edit distance and reconstructed mutational histories relative to the unmutated reference germline by successively adding each sequence to the least distant sequence in the network. Investigating the mutational network containing the lysate‐binding BM PC demonstrated 17 distinct antibody sequences with varying degrees of clonal expansion, isotype distribution, and transcriptional phenotype (Fig. 4D, Supporting information Fig. S9A). Comparing the differentially expressed genes between cells from the lysate‐specific clone (defined by unique CDRH3 + CDRL3 AA sequence) and the virus‐specific clones demonstrated only four differentially expressed genes (adjusted *p* value < 0.01) compared to PCs with GPC‐ or NP‐specific antibodies (Fig. 4E, Supporting information Tables S7 and S8). There were no indications of enrichment of genes involved in autoreactivity when performing an unbiased gene ontology analysis (Supporting information Fig. S9B and 9C), which could be expected given the minor transcriptional differences to the virus‐specific clones. Next, we determined whether this clone would demonstrate specificity to various murine tissue lysates as it did to the murine MC57G fibroblast cell‐line lysate. We performed ELISA against lysates from various organs of uninfected mice. Using both commercial and self‐prepared lysates, we could not detect any reactivity relative to the positive control antiactin antibody (Fig. 4F, Supporting information Fig. S9D), highlighting the difficulty of discovering the target present in both naive and infected lysate for this clonally expanded IgG antibody.

### Distinct repertoire and transcriptional features of GPC and NP‐specific plasma cells

**Figure 5:**
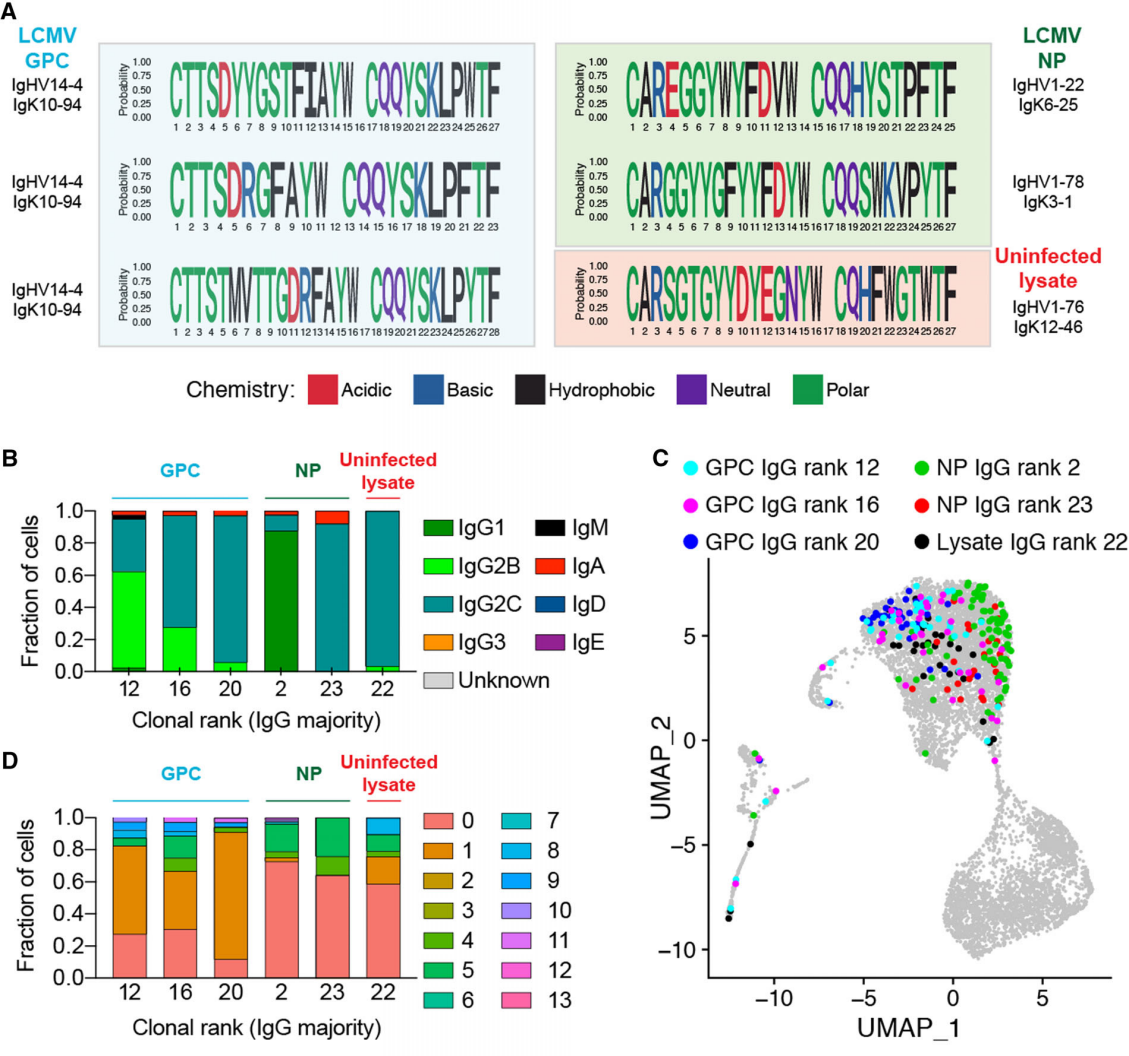
Repertoire and transcriptome profile of LCMV‐specific PCs. (A) Sequence logo plots of the confirmed GPC, NP, and MC57G lysate binders. (B) Isotype distribution for the confirmed GPC, NP, and MC57G lysate binders. (C) Location of the confirmed GPC, NP, and MC57G lysate binders on the UMAP. (D) Transcriptome distribution for the confirmed GPC, NP, and MC57G lysate binders. Color indicates the transcriptional cluster from 3A.

We next characterized the repertoire features involving germline gene usage, sequence motifs, isotype for the GPC‐ and NP‐specific clones. For the three antibody clones that had specificity to GPC, a convergence toward the same variable germline gene combination (IgHV14‐4, IgKV10‐94) was observed, despite the clones having different CDRH3 + CDRL3 AA sequences and lengths (Fig. 5A. Further investigation into the V gene regions of the three GPC‐specific antibodies demonstrated 12 AA mutations across either the V_H_ or V_L_ segments despite convergent germline gene usage and CDR3 sequence (Supporting information Fig. S10). Conversely, the NP and uninfected lysate antibodies used distinct germline genes and had more biochemical variation in their CDRH3 regions compared to the GPC binders (Fig. 5A). Analyzing the isotype distribution demonstrated IgG subtype heterogeneity across the GPC‐specific clones despite the identical germline gene usage and similar CDR3s (Fig. 5B). Visualizing these selected clones using UMAP based on their transcriptomes suggested that the NP‐ and GPC‐specific clones occupied transcriptionally distinct states, and quantifying their cluster membership showed they primarily belonged to either cluster 0 or cluster 1, respectively (Fig. 5C and D). Differential gene expression analysis revealed that IFN‐related genes, such as *Ifnar2* and *Ifitm3*, were upregulated in cluster 0 whereas genes, such as *Tigit*, *CD24a*, were upregulated in cluster 1 (Supporting information Fig. S11A, Table S9). Performing an additional differential expression analysis of all cells binding LCMV revealed that GPC‐specific clones significantly upregulated MHC‐II genes compared to the NP‐specific clones, which in turn, demonstrated increased IFN‐related genes (Supporting information Fig. S11B, Table S10). Taken together, these findings support a model in which plasma cells targeting different viral proteins occupy distinct transcriptional states.

### Virus‐specific plasma cells gain cross‐reactivity via somatic hypermutation

**Figure 6:**
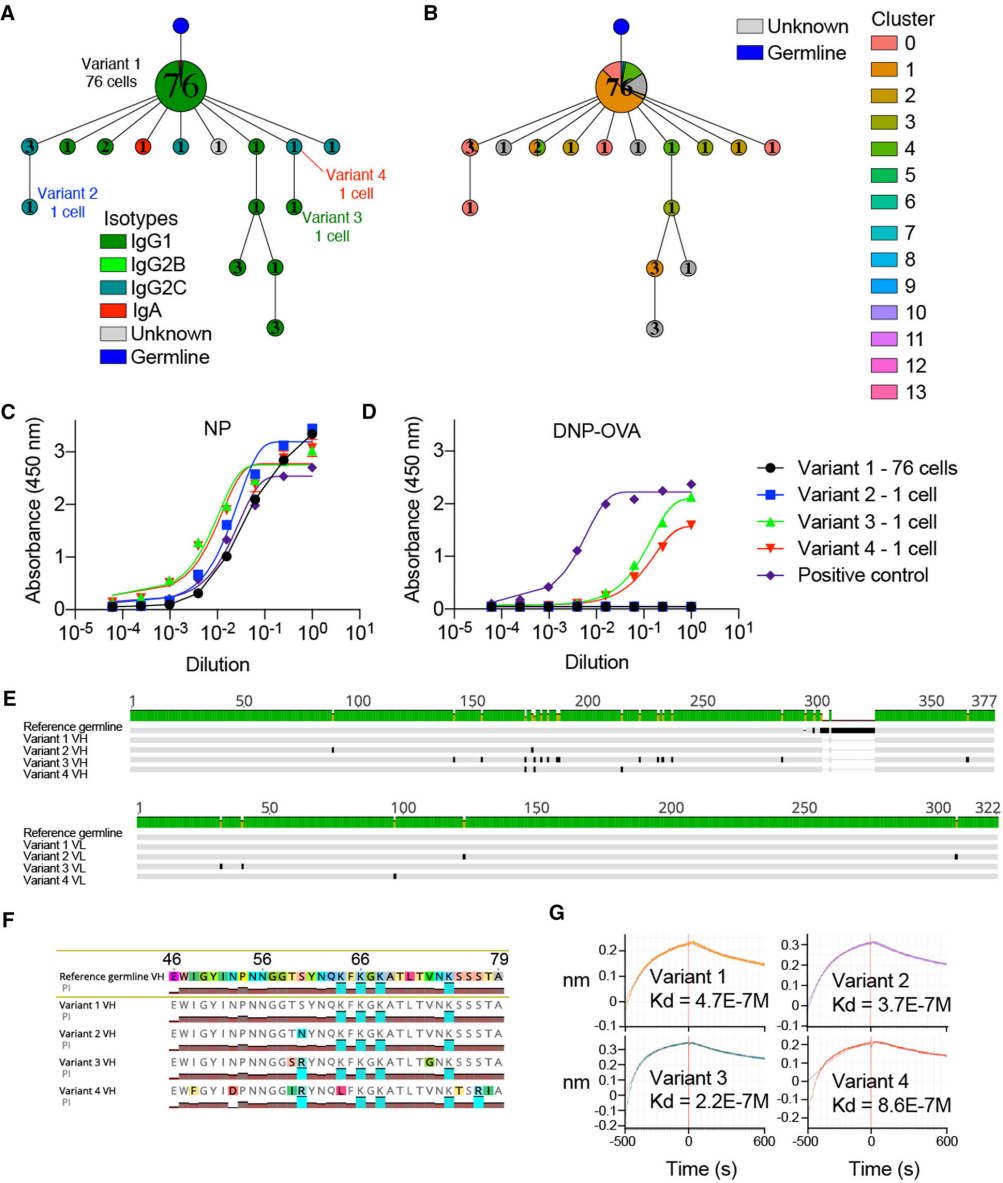
Virus‐specific somatic variants present in the BM PC repertoire are cross‐reactive. (A,B) Mutational network of the NP‐specific, second most expanded IgG clone. Nodes represent unique antibody variants (combined V_H_+V_L_ nucleotide sequence) and edges demonstrate sequences with the smallest separation calculated by edit distance. Node color corresponds to either isotype (A) or transcriptional cluster (B). The size and label of the nodes indicate how many cells express each full‐length antibody variant. Clone was determined by grouping those B cells containing identical CDRH3 + CDRL3 AA sequences. Only cells containing exactly one variable heavy (V_H_) and variable light (V_L_) chain were considered. The isotype was determined as the isotype corresponding to the majority of cells within one clone. Variant labels indicate those full‐length antibodies which were recombinantly expressed. The germline node represents the unmutated reference sequence determined by 10× Genomics cellranger. (C) NP ELISA for the four variants from the second most expanded IgG clone. (D) DNP‐OVA ELISA for the four variants from the second most expanded IgG clone. (E) V_H_ and V_L_ nucleotide alignments of the four variants from the second most expanded IgG clone. (F) V_H_ amino acid alignment highlighting mutations in the CDRH2. Colored graphs correspond to isoelectric point (PI). (G) Affinity measurements (*k_d_*) against LCMV NP for the four variants from the second most expanded IgG clone in molar (M) concentrations.

Since, we were unable to determine the specificity of the antibody clone recognizing lysate of uninfected MC57G cells, we shifted our analysis to those clones with confirmed LCMV specificity. We first analyzed the relationship between somatic hypermutation, clonal expansion, and transcriptional phenotype of specific clonally related antibody variants within virus‐specific clonal lineages. Inferring mutational networks for the remaining virus‐specific clonal lineages demonstrated heterogeneity across the distance of clonally expanded antibody variants from germline, isotype distribution, and transcriptional cluster membership (Fig. 6A and B, Supporting information Fig. S12, S13). Highlighting the lineage containing the second most expanded clone, which we previously determined as NP‐specific (Fig. 4A, Supporting information Fig. S8), revealed that the most expanded antibody variant (which we had also tested) was closest to the germline, predominantly of the IgG1 isotype, and located primarily in transcriptional cluster 0 (Fig. 6A and B).

We next addressed whether the mutated variant antibodies produced by unexpanded BM PCs maintained reactivity to NP. We, therefore, additionally expressed three variants that had incurred somatic hypermutations across both V_H_ and V_L_ chain sequences relative to both the unmutated germline reference sequence and the most expanded BM PC variant in the clonal lineage with confirmed NP specificity (Figs. 4A and 6C and Supporting information Fig. S8). ELISA confirmed that all three tested variants maintained NP specificity with comparable signals to the previously tested variant and our positive control antibody (Fig. 6C, Supporting information Fig. S14). Surprisingly, when screened for specificity against other antigens (LCMV GPC, insulin, dsDNA, DNP‐OVA, lysate of uninfected and infected cells), we discovered that two of the variants specifically bound DNP‐OVA but not the other tested LCMV‐unrelated antigens (Fig. 6D, Supporting information Fig. S14), thereby, providing a potential explanation for the LCMV‐unspecific IgG antibody response characteristic of chronic viral infection [8]. Sequence analysis revealed that the three variants had incurred multiple somatic hypermutations, preferentially in their CDRH2 relative to the most expanded variant and reference germline (Fig. 6E). Closer inspection revealed that the two cross‐reactive variants shared a common S60R mutation in the Vernier region of the CDRH2 relative to the other two single‐reactive antibodies (Fig. 6F), further supporting the hypothesis that cross‐reactivity was acquired by somatic hypermutations and CDRH2 loop flexibility. We lastly determined whether acquiring cross‐reactivity reduced the affinity against LCMV NP by measuring the equilibrium dissociation constants (*K_d_*) for the four variants. Interestingly, the *K_d_* values were relatively low for all four variants, with no trend that cross‐reactivity diminished NP‐binding ability (Fig. 6G).

## Discussion

Determining the specificity of antibodies produced by plasma cells, residing mainly in the BM, is challenging due to the reduced surface expression of the BCR [13, 16]. Even though there have been significant advances recently to address this problem [18, 25, 26, 27], most of the previous studies have been reliant upon bulk assays to quantify circulating serum antibodies (ELISA) or antibody‐secreting cells (ELISpot). These classical approaches fail to inform about the genetics and the clonal diversity of a virus‐specific antibody response (i.e., how many unique antibody clones), and about the viral protein specificity of individual BM PCs. Our integrated scSeq approach resolved this short coming, as we were able to recover thousands of naturally paired V_H_ and V_L_ sequences and their corresponding isotypes, some of which were represented across a multitude of distinct PCs (Fig. 1). Overall, we observed differences in isotype distribution, clonal expansion, somatic hypermutation, and gene expression profiles when comparing chronic viral infection to protein immunizations (Figs. 1, 2, 3, Supporting information Fig. S1). Although these findings need to be interpreted with caution, it nevertheless provided an initial insight into potential differences of identity adopted by BM PCs arising in the context of a chronic viral infection as compared to BM PCs developing after protein immunization.

With the recent advent of scSeq workflows, it is now possible to comprehensively test and reconstruct the specificity of BM PCs based on their clonal expansion profiles. Using high‐throughput antibody expression and screening, we were able to demonstrate that a fraction of expanded cells in the BM PC compartment produce antibodies against LCMV‐derived NP and GPC antigens following chronic LCMV infection (Fig. 4). Given the high abundance of NP protein compared to other viral proteins [28, 29] in infected cells, it seems intuitive that several of the highest expanded antibody clones were specific against this viral target. We cannot rule out, however, that the viral proteins present at 28 dpi have acquired escape mutations compared to the purified WT NP/GPC protein as well as the LCMV clone 13 virus cell lysate that was used for experimental validation, such that antibody binding to these potential variants was not detected in the applied assays. Despite these screening limitations, we were able to demonstrate that during chronic viral infection, somatic hypermutation was implicated in the evolution of viral‐specific antibodies into cross‐reactive antibodies with specificity to unrelated nonviral antigens (Fig. 6). Chronic HIV infection has also been implicated in the development of cross‐specificity of HIV‐specific antibodies toward other antigens (i.e., phospholipids) [30, 31], however, the extent of this cross‐reactive capacity within a given antigen‐specific clonal lineage has not been determined. However, we could further demonstrate in this study that the cross‐reactive BM PCs were not clonally expanded within the clonal family (Fig. 6), implying that acquiring cross‐reactivity does not coincide with a selective advantage, as we would otherwise expect the most expanded antibody variant to demonstrate cross‐reactivity. Furthermore, we discovered a clonally expanded IgG class‐switched and mutated clone that showed high reactivity to both lysates of uninfected as well as infected MC57G cells (Fig. 4, Supporting information Figs. S8, S9) implying autoreactive capacity as has been reported previously for high‐dose LCMV infection [9, 10]. Although this cancerous fibroblast cell line originates from a C57BL/6 mouse, we could not demonstrate binding to protein extracts from various tissue lysates from uninfected C57BL/6 mice or other known targets for autoreactive clones such as insulin or dsDNA (Supporting information Fig. S8). To determine the binding antigen as well as tissue specificity, coimmunoprecipitation followed by tandem MS or serological analysis of expression cDNA libraries (SEREX) [10, 32] could be used in future studies.

Our data suggest that there may be stereotypical binding preferences for GPC, but not NP reactive clones, as the GPC binding clones utilized identical germline gene combinations and similar CDR3 sequences (Fig. 4) [33]. In contrast, the NP‐specific BM PCs utilized different germline genes (Fig. 5), consistent with previous V_H_ repertoire sequencing results, which demonstrated a continuous recruitment of unique clones into the BM PC repertoire over time during chronic but not acute LCMV infection [13]. Although our study relies on the BM PC compartment of a single infected mouse, we have nevertheless demonstrated that our scSeq pipeline can be useful in profiling a personalized, virus‐specific plasma cell response.

Finally, our study linked antibody repertoire information with total transcriptome profiles, providing a detailed transcriptional description of distinct plasma cell clonotypes in the BM. Our results suggest that IgM‐ and IgA‐secreting BM PCs have distinct transcriptional profiles compared to the IgG clones, and that within virus‐specific IgG clones there was transcriptional diversity (Fig. 5). Some antibody discovery pipelines rely heavily on class‐switched IgG memory B cells following immunization schemes [34], leveraging the ability to directly stain surface BCRs and identify antigen‐specific B cells via flow cytometry. The lack of surface BCR/antibody on BM PCs means that a scSeq approach was required to interrogate binding specificity. This also allowed us to pair transcriptome information with antibody sequence which further revealed that BM PCs producing either LCMV NP‐ or GPC‐specific antibodies had distinct transcriptional profiles (Fig. 5). While this analysis was restricted to approximately 100 cells for both antigens, it nevertheless demonstrated an increase of MHC‐II gene expression in the GPC‐specific BM PCs, in contrast to, an increase of inflammatory IFN‐induced genes in the NP‐specific BM PCs (Fig. 5). This transcriptional program may reflect distinct selection pressures for GPC‐ in comparison to NP‐specific BM PCs at this time point, thereby promoting the onset of neutralizing antibodies. Future studies analyzing GPC and NP‐specific BM PCs at early and later time points could better delineate whether such a transcriptional phenotype is exclusively reserved for GPC‐specific BM PCs, or reflects longitudinal GC selection based on which viral protein complexes are displayed and recognized on follicular DCs.

### Study limitations

The results presented in this study require caution when interpreting and extrapolating findings relating to fundamental plasma cell biology. One major concern relates to that infection and immunization strategies contained only a single mouse per condition. Experimental variability between individual animals may dictate the reported differences relating to both repertoire features and gene expression phenotypes. One prominent example is the sample‐specific clustering of PCs following either immunization with TNFR2 or chronic LCMV infection (Fig. 3), as these results are likely influenced by batch effects arising from different experimental conditions. Although we normalized for the total number of counts in each cell (following filtering immune receptor genes), we cannot exclude that the isotype‐specific and sample‐specific transcriptional differences were impacted by removing immune receptor transcripts. Similarly, the strain and sex of the mice varied across the experimental conditions, undoubtedly influencing the comparative results. Although we have included two distinct immunization conditions to provide context to the PC repertoire following viral infection, we cannot yet conclude the robustness of these findings. These experimental differences could additionally influence the isotype specific properties of the repertoires that we documented, some of which may be entirely unrelated to the induced infection or immunizations. Therefore, we focused our analysis on the clonally expanded and antigen‐specific IgG PCs following chronic LCMV infection, as these cells arise from a single sequencing experiment and repertoire features (e.g., transcriptional signatures between GPC and NP‐specific PCs or antibody isotypes) should not be influenced by batch effects. Nevertheless, we acknowledge that our transcriptional findings are based on a low number of cells and clones, which may also contribute to the observed protein‐specific gene expression profiles. Future studies, including larger cohorts and a higher number of antigen‐specific PCs, are required to corroborate our presented hypotheses. Finally, profiling repertoire features and antigen specificity of plasma cell repertoires in naïve mice would help elucidate which affects viral infection had on the selection of our reported virus‐specific antibodies. Although previous results of naïve mice from our animal facility indicate undetectable titers to LCMV proteins and DNP‐OVA, it would be nevertheless of interest to profile whether such virus‐specific antibodies exist independent of infection.

## Materials and methods

### Mouse experiments

All animal experiments were performed in accordance with institutional guidelines and Swiss federal regulations. Experiments were approved by the veterinary office of the canton of Zurich (animal experimentation permissions 115/2017) and Basel‐Stadt (animal experimentation permission 2582). A 6‐ week‐old female C57BL/6 mouse was infected with 2 × 10^6^ LCMV clone 13 intravenously as previously described [13] and sacrificed at 28 dpi. LCMV clone 13 was propagated by infection of BHK‐21 cells for 24–48 h and then resuspended in sterile PBS (GIBCO).

For protein immunizations, a 6‐week‐old male C57BL/6 mouse was repeatedly immunized five times every other week s.c. into the flank with 10 μg of human TNFR2 protein (Peprotech, 310–12)/20 μg MPLA (Sigma, L6895) adjuvant and sacrificed 1 week afterwards. Likewise, a 6‐week‐old female BALB/c mouse was injected three times with 4 and 3 weeks intervals s.c. into the flank with 100 μg of OVA (Sigma, A5503)/20 μg MPLA (Sigma, L6895) adjuvant and sacrificed 2 weeks after the final boost.

### Isolation of BM plasma cells

A single‐cell suspension was prepared by flushing the BM from the tibia, femur, and hip bones in RPMI containing 10% FCS buffer with 10 ng/mL IL6 (Peprotech, 216‐16). An RBC lysis step was performed in 4 mL ammonium‐chloride‐potassium lysis buffer for 1 min at room temperature and subsequently inactivated with 26 mL RPMI containing 10% FCS. For LCMV infection and TNFR2 immunizations, single‐cell suspensions were stained with the following antibodies (1:200 dilution) CD138‐APC, CD4‐APC‐Cy7, CD8a‐APC‐Cy7, NK1.1‐APCCy7, Ter119‐APC‐Cy7, TACI‐PE, B220‐APC, CD19‐PE‐Cy7 for 30 minutes at 4°C. Cell sorting was performed using a FACSAria with FACSDiva software into complete RPMI. For the OVA immunized mouse, BM PCs were isolated using a CD138+ plasma cell isolation kit (Miltenyi, 130‐092‐530) following the instructions of the manufacturer.

### Library construction and sequencing

Single‐cell 10× VDJ libraries were constructed from the isolated BM plasma cells following the demonstrated protocol “Direct target enrichment —Chromium Single Cell V(D)J Reagent Kits” (CG000166 REV A). Briefly, single cells were coencapsulated with gel beads (10× Genomics, 1000006) in droplets using three lanes of one Chromium Single‐Cell A Chip (10× Genomics, 1000009) with a target loading of 13,000 cells per reaction. V(D)J library construction was carried out using the Chromium Single Cell 5’ Library Kit (10× Genomics, 1000006) and the Chromium Single Cell V(D)J Enrichment Kit, Mouse B Cell (10× Genomics, 1000072). Final libraries were pooled and sequenced on the Illumina NextSeq 500 platform (mid output, 300 cycles, paired‐end reads) using an input concentration of 1.8 pM with 5% PhiX.

### Repertoire and transcriptome analysis

Paired‐end raw sequencing files from Illumina NextSeq500 run were aligned to murine reference genome and germlines (GRCm38) using 10× Genomics cellranger (v4.0.1) with clonotyping based on those B cells containing identical CDRH3 + CDRL3 nucleotide sequences. Gene expression analysis was performed using the R package Seurat (version 3.6) [35] using the automate_GEX function from the R package Platypus (version 2.5) under default parameters [36]. Specifically, those cells lacking full‐length, paired (V_H_+V_L_) antibody sequences in the VDJ library were removed from the transcriptome analysis. Furthermore, genes dictating clonal diversity (e.g., IgH V genes, constant genes, IgH J genes) were removed from the gene expression matrix where indicated. Cells containing more than 5% mitochondrial genes were filtered out before log‐normalization using a scaling factor of 10,000. Mean expression and variance were further scaled to 0 and 1, respectively. A total of 2000 variable features were selected using the “vst” selection method and used as input to PCA. Graph‐based clustering incorporating Louvain modularity optimization and hierarchical clustering was performed by the Seurat functions FindNeighbors and FindClusters using the first 10 dimensions and a cluster resolution of 0.5 as suggested by the developers [35]. UMAP was calculated using the first 10 dimensions. When calculating clonal frequencies and performing all differential gene analyses, those clones sharing identical CDRH3 + CDRL3 AA sequences were clustered into a single clonal family. Full‐length sequences ranging from framework region 1 to framework region 4 were annotated using the built‐in murine reference alleles in MiXCR and exported by the VDJRegion gene feature (v3.0.1). The full‐length, heavy and light VDJRegion sequences corresponding to identical cellular barcodes were appended and then the number of clonal variants was determined by calculating the number of unique AA sequences. These full‐length, appended V_H_+V_L_ sequences were subsequently used to create mutational networks, which were constructed by first clustering all B cells containing identical V and J segments for heavy and light chain, identical CDRH3 and CDRL3 sequence lengths, and sharing 70% sequence homology. The pairwise edit distance was subsequently calculated for each full‐length VDJ sequence (including the reference germline), generating a distance matrix to determine the order in which sequences were added to the network. The unmutated germline reference gene (determined by cellranger alignments) initialized each network, and then the sequence with the smallest distance was added to the most similar sequence in the network iteratively. Edit distance ties were resolved by randomly sampling all possible nodes. The final adjacency matrix was visualized as a graph by using the graph_from_adjacency_matrix function in the R package igraph [37]. Cluster‐specific genes were determined by Seurat's FindAllMarkers function and were subsequently ranked by average log fold‐change. Differential gene analysis for all comparisons was performed using the the Wilcoxon Rank Sum test in the FindMarkers function in Seurat with the logfc.threshold set to 0.25 with clonal definition as CDRH3 + CDRL3 AA sequence. Cell isotype for gene expression data was extracted by matching barcodes from the VDJ sequencing data. Multiple string alignments and visualizations were performed using Geneious. Gene ontology analysis was performed using the function goanna in the R package edgeR under default parameters [38]. Heatmaps, feature plots, and violin plots were produced by supplying genes of interest to the functions DoHeatmap, FeaturePlot, and VlnPlot functions in Seurat, respectively.

### Antibody expression and validation

Mouse antibodies were produced as previously described [23] and validated using normalized supernatant ELISAs against purified NP, GPC, and DNP‐OVA, insulin (Sigma, I5500), mouse genomic DNA (Sigma, 692339), as well as in house‐produced lysate of (LCMV‐infected) MC57G cells as previously described [3]. Recombinant LCMV clone 13 NP and GPC protein were expressed as previously described [3, 39]. An anti‐mouse IgG‐HRP (Sigma, A2554) was employed at 1:1500 and used for detection. To test for tissue specificity of the autoreactive clone, protein extracts from commercial BM tissue (Zyagen, MT‐704‐C57) as well as self‐made extracts from spleen and BM were tested at a 100 μg/mL coating concentration. Self‐made extracts were prepared from spleen and BM single‐cell suspensions. Cells were centrifuged 5 min at 1600 rpm at 4°C and the pellet was resuspended in 5 mL of PBS. Cells were lysed using a syringe (28G needle), homogenizing several times and additionally sonicated (three times 20 s at 40 MHz) on ice. Anti‐HEL antibody (in house) as well as anti‐mouse beta‐tubulin IgG (Sigma, T5201), anti‐mouse beta‐actin IgG (Sigma, A2228), anti‐OVA IgG (in house), anti‐NP‐IgG Pank1, anti‐GPC‐IgG Wen1.3, anti‐insulin IgG E11D7 (Sigma, 05–1066), and anti‐dsDNA IgG AE‐2 (Sigma, MAB1293) were used as negative and positive controls for respective experiments and employed at 4 μg/mL.

### Antibody affinity measurements

Antibody affinity measurements were carried out as reported previously [23, 40]. In brief, biolayer interferometry assays were performed on the Octet Red96 (ForteBio) at 25˚C, shaking at 1000 rpm. Kinetics assays were performed with anti‐mouse IgG Fc Capture (AMC) Biosensors (ForteBio, Cat‐No. 18–5090) with the following steps: (0) Hydration of AMC Biosensors in 1× kinetics buffer for 30 min (ForteBio, Cat‐No. 18–1105). (1) Baseline equilibration in conditioned medium diluted 1:1 with 1× kinetics buffer for 60 s. (2) Regeneration of sensors (3×) in 10 mM Glycine. (3) Baseline for 300 s. (4) Loading of antibodies contained in supernatant for 500 s. (5) Quenching/blocking of sensors in 50 μg/mL hIgG in 1× KB for 30 s. (6) Antigen association: sensors immersed with NP at 3.2–2000 nM for 500 s. (7) Dissociation in 1× KB for 600 s. (8) Regeneration of sensors. Curve fitting was performed using the ForteBio Octet HTX data analysis software using a 1:1 model, and a baseline correction using a reference sensor.

#### Data visualization

Heatmaps displaying differential gene expression were produced using the DoHeatmap function in the R package Seurat [41]. Gene enrichment plots were produced using the R package ggplot [42]. Mutational networks were produced using the R package igraph [37]. Sequence logo plots were generated using the R package ggseqlogo [43]. Sequence alignment plots were exported from Geneious Prime. All other figures were produced using Prism v9 (Graphpad), excluding the graphical abstract, which was created with BioRender.com.

## Author contributions

DN, AP, AG, IS, RE, KH, LH, LC, and AY performed experiments. DN, IS, CP, AA, RK, DR, JH, and AY performed computational analysis. All authors contributed to the manuscript and study design.

## Funding

This work was supported by the European Research Council Starting Grant 679403 (to STR), ETH Zurich Research Grants (to STR and AO), and an ETH Career Seed Grant (to AY).

### Peer review

The peer review history for this article is available at https://publons.com/publon/10.1002/eji.202149331

## Conflict of interest

Authors declare that there are no conflict of interest.

**Abbreviations**
DNP: dinitrophenol
dpi: days post infection
GPC: glycoprotein complex
LCMV: Lymphocytic choriomeningitis virus
NP: nucleocapsid protein
PHGG: polyclonal hypergammaglobulinemia
scSeq: single‐cell sequencing
UMAP: uniform manifold approximation projection

## Supporting information



Supporting Information

Table S1

Table S2

Table S3

Table S4

Table S5

Table S6

Table S7

Table S8

Table S9

Table S10

## Data Availability

Both the raw sequencing files and alignment outputs from deep sequencing that support the findings of this study have been deposited in the European Bioinformatics Institute under accession number E‐MTAB‐11096. Additional data that support the findings of this study are available from the corresponding author.

## References

[1] Bootz, A. , Karbach, A. , Spindler, J. , Kropff, B. , Reuter, N. , Sticht, H. , Winkler, T. H. et al., Protective capacity of neutralizing and non‐neutralizing antibodies against glycoprotein B of cytomegalovirus. PLoS Pathog. 2017. 13: e1006601. 28854233 10.1371/journal.ppat.1006601 PMC5595347

[2] Burton, D. R. and Hangartner, L. , Broadly neutralizing antibodies to HIV and their role in vaccine design. Annu. Rev. Immunol. 2016. 34: 635–659. 27168247 10.1146/annurev-immunol-041015-055515 PMC6034635

[3] Greczmiel, U. , Kräutler, N. J. , Pedrioli, A. , Bartsch, I. , Agnellini, P. , Bedenikovic, G. , Harker, J. et al., Sustained T follicular helper cell response is essential for control of chronic viral infection. Sci. Immunol. 2017. 2: eaam8686. 29196449 10.1126/sciimmunol.aam8686

[4] Nguyen, D. C. , Joyner, C. J. , Sanz, I. and Lee, F. E. ‐H. , Factors affecting early antibody secreting cell maturation into long‐lived plasma cells. Front. Immunol. 2019. 10: 2138. 31572364 10.3389/fimmu.2019.02138 PMC6749102

[5] Battegay, M. , Moskophidis, D. , Waldner, H. , Bründler, M. A. , Fung‐Leung, W. P. , Mak, T. W. , Hengartner, H. et al., Impairment and delay of neutralizing antiviral antibody responses by virus‐specific cytotoxic T cells. J. Immunol. 1993. 151: 5408–5415. 7693811

[6] Hangartner, L. , Senn, B. M. , Ledermann, B. , Kalinke, U. , Seiler, P. , Bucher, E. , Zellweger, R. M. et al., Antiviral immune responses in gene‐targeted mice expressing the immunoglobulin heavy chain of virus‐neutralizing antibodies. Proc. Natl. Acad. Sci. U. S. A. 2003. 100: 12883–12888. 14569006 10.1073/pnas.2135542100 PMC240713

[7] Kimmig, B. and Lehmann‐Grube, F. , The immune response of the mouse to lymphocytic choriomeningitis virus. I. Circulating antibodies. J. Gen. Virol. 1979. 45: 703–710. 541676 10.1099/0022-1317-45-3-703

[8] Greczmiel, U. , Kräutler, N. J. , Borsa, M. , Pedrioli, A. , Bartsch, I. , Richter, K. , Agnellini, P. et al., LCMV‐specific CD4 T cell dependent polyclonal B‐cell activation upon persistent viral infection is short lived and extrafollicular. Eur. J. Immunol. 2020. 50: 396–403. 31724162 10.1002/eji.201948286 PMC7079077

[9] Hunziker, L. , Recher, M. , Macpherson, A. J. , Ciurea, A. , Freigang, S. , Hengartner, H. and Zinkernagel, R. M. , Hypergammaglobulinemia and autoantibody induction mechanisms in viral infections. Nat. Immunol. 2003. 4: 343–349. 12627229 10.1038/ni911

[10] Ludewig, B. , Krebs, P. , Metters, H. , Tatzel, J. , Türeci, O. and Sahin, U. , Molecular characterization of virus‐induced autoantibody responses. J. Exp. Med. 2004. 200: 637–646. 15353556 10.1084/jem.20040358 PMC2212735

[11] Manz, R. A. , Thiel, A. and Radbruch, A. , Lifetime of plasma cells in the bone marrow. Nature 1997. 388: 133–134. 9217150 10.1038/40540

[12] Halliley, J. L. , Tipton, C. M. , Liesveld, J. , Rosenberg, A. F. , Darce, J. , Gregoretti, I. V. , Popova, L. et al., Long‐lived plasma cells are contained within the CD19(−)CD38(hi)CD138(+) subset in human bone marrow. Immunity 2015. 43: 132–145. 26187412 10.1016/j.immuni.2015.06.016 PMC4680845

[13] Kräutler, N. J. , Yermanos, A. , Pedrioli, A. , Welten, S. P. M. , Lorgé, D. , Greczmiel, U. , Bartsch, I. et al., Quantitative and qualitative analysis of humoral immunity reveals continued and personalized evolution in chronic viral infection. Cell Rep. 2020. 30: 997–1012.e6. 31995768 10.1016/j.celrep.2019.12.088

[14] Medina, F. , Segundo, C. , Campos‐Caro, A. , González‐García, I. and Brieva, J. A. , The heterogeneity shown by human plasma cells from tonsil, blood, and bone marrow reveals graded stages of increasing maturity, but local profiles of adhesion molecule expression. Blood 2002. 99: 2154–2161. 11877292 10.1182/blood.v99.6.2154

[15] Painter, S. D. , Haralambieva, I. H. , Ovsyannikova, I. G. , Grill, D. E. and Poland, G. A. , Detection of influenza A/H1N1‐specific human IgG‐secreting B cells in older adults by ELISPOT assay. Viral Immunol. 2014. 27: 32–38. 24605786 10.1089/vim.2013.0099 PMC3949448

[16] Wolf, A. I. , Mozdzanowska, K. , Quinn, W. J. , 3rd , Metzgar, M. , Williams, K. L. , Caton, A. J. , Meffre, E. et al., Protective antiviral antibody responses in a mouse model of influenza virus infection require TACI. J. Clin. Invest. 2011. 121: 3954–3964. 21881204 10.1172/JCI57362 PMC3195469

[17] Croote, D. , Darmanis, S. , Nadeau, K. C. and Quake, S. R. , High‐affinity allergen‐specific human antibodies cloned from single IgE B cell transcriptomes. Science 2018. 362: 1306–1309. 30545888 10.1126/science.aau2599

[18] Horns, F. , Dekker, C. L. and Quake, S. R. . , Memory B cell activation, broad anti‐influenza antibodies, and bystander activation revealed by single‐cell transcriptomics. Cell Rep. 2020. 30: 905–913.e6. 31968262 10.1016/j.celrep.2019.12.063 PMC7891556

[19] Saikia, M. , Burnham, P. , Keshavjee, S. H. , Wang, M. F. Z. , Heyang, M. , Moral‐Lopez, P. , Hinchman, M. M. et al., Simultaneous multiplexed amplicon sequencing and transcriptome profiling in single cells. Nat. Methods 2019. 16: 59–62. 30559431 10.1038/s41592-018-0259-9 PMC6378878

[20] Singh, M. , Al‐Eryani, G. , Carswell, S. , Ferguson, J. M. , Blackburn, J. , Barton, K. , Roden, D. et al., High‐throughput targeted long‐read single cell sequencing reveals the clonal and transcriptional landscape of lymphocytes. Nat. Commun. 2019. 10: 3120. 31311926 10.1038/s41467-019-11049-4 PMC6635368

[21] Pracht, K. , Meinzinger, J. , Daum, P. , Schulz, S. R. , Reimer, D. , Hauke, M. , Roth, E. et al., A new staining protocol for detection of murine antibody‐secreting plasma cell subsets by flow cytometry. Eur. J. Immunol. 2017. 47: 1389–1392. 28608550 10.1002/eji.201747019

[22] Barnett, B. E. , Staupe, R. P. , Odorizzi, P. M. , Palko, O. , Tomov, V. T. , Mahan, A. E. , Gunn, B. et al., Cutting edge: B cell‐intrinsic T‐bet expression is required to control chronic viral infection. J. Immunol. 2016. 197: 1017–1022. 27430722 10.4049/jimmunol.1500368 PMC4975981

[23] Parola, C. , Neumeier, D. , Friedensohn, S. , Csepregi, L. , Di Tacchio, M. , Mason, D. M. and Reddy, S. T. , Antibody discovery and engineering by enhanced CRISPR‐Cas9 integration of variable gene cassette libraries in mammalian cells. MAbs 2019. 11: 1367–1380. 31478465 10.1080/19420862.2019.1662691 PMC6816377

[24] Pogson, M. , Parola, C. , Kelton, W. J. , Heuberger, P. and Reddy, S. T. , Immunogenomic engineering of a plug‐and‐(dis)play hybridoma platform. Nat. Commun. 2016. 7: 12535. 27531490 10.1038/ncomms12535 PMC4992066

[25] Eyer, K. , Doineau, R. C. L. , Castrillon, C. E. , Briseño‐Roa, L. , Menrath, V. , Mottet, G. , England, P. et al., Single‐cell deep phenotyping of IgG‐secreting cells for high‐resolution immune monitoring. Nat. Biotechnol. 2017. 35: 977–982. 28892076 10.1038/nbt.3964

[26] Gérard, A. , Woolfe, A. , Mottet, G. , Reichen, M. , Castrillon, C. , Menrath, V. , Ellouze, S. et al., High‐throughput single‐cell activity‐based screening and sequencing of antibodies using droplet microfluidics. Nat. Biotechnol. 2020. 38: 715–721. 32231335 10.1038/s41587-020-0466-7

[27] Setliff, I. , Shiakolas, A. R. , Pilewski, K. A. , Murji, A. A. , Mapengo, R. E. , Janowska, K. , Richardson, S. et al., High‐throughput mapping of B cell receptor sequences to antigen specificity. Cell 2019. 179: 1636–1646.e15. 31787378 10.1016/j.cell.2019.11.003 PMC7158953

[28] King, B. R. , Samacoits, A. , Eisenhauer, P. L. , Ziegler, C. M. , Bruce, E. A. , Zenklusen, D. , Zimmer, C. et al., Visualization of arenavirus RNA species in individual cells by single‐molecule fluorescence in situ hybridization suggests a model of cyclical infection and clearance during persistence. J. Virol. 2018. 92: e02241‐e02517 10.1128/JVI.02241-17 PMC5974494 29643234

[29] Pinschewer, D. D. , Perez, M. and de la Torre, J. C. , Role of the virus nucleoprotein in the regulation of lymphocytic choriomeningitis virus transcription and RNA replication. J. Virol. 2003. 77: 3882–3887. 12610166 10.1128/JVI.77.6.3882-3887.2003 PMC149515

[30] Haynes, B. F. , Fleming, J. , St Clair, E. W. , Katinger, H. , Stiegler, G. , Kunert, R. , Robinson, J. et al., Cardiolipin polyspecific autoreactivity in two broadly neutralizing HIV‐1 antibodies. Science 2005. 308: 1906–1908. 15860590 10.1126/science.1111781

[31] Matyas, G. R. , Beck, Z. , Karasavvas, N. and Alving, C. R. , Lipid binding properties of 4E10, 2F5, and WR304 monoclonal antibodies that neutralize HIV‐1. Biochim. Biophys. Acta 2009. 1788: 660–665. 19100711 10.1016/j.bbamem.2008.11.015

[32] Chen, Y. ‐T. , Gure, A. O. and Scanlan, M. J. , Serological analysis of expression cDNA libraries (SEREX). In Su, G. H. (Ed.), Pancreatic cancer: methods and protocols, Humana Press, Totowa, NJ, 2005, pp. 207–216. 15542909

[33] Hsiao, Y. ‐C. , Chen, Y. ‐J. J. , Goldstein, L. D. , Wu, J. , Lin, Z. , Schneider, K. , Chaudhuri, S. et al., Restricted epitope specificity determined by variable region germline segment pairing in rodent antibody repertoires. MAbs 2020. 12: 1722541. 32041466 10.1080/19420862.2020.1722541 PMC7039645

[34] Parola, C. , Neumeier, D. and Reddy, S. T. , Integrating high‐throughput screening and sequencing for monoclonal antibody discovery and engineering. Immunology 2018. 153: 31–41. 28898398 10.1111/imm.12838 PMC5721244

[35] Satija, R. , Farrell, J. A. , Gennert, D. , Schier, A. F. and Regev, A. , Spatial reconstruction of single‐cell gene expression data. Nat. Biotechnol. 2015. 33: 495–502. 25867923 10.1038/nbt.3192 PMC4430369

[36] Yermanos, A. , Agrafiotis, A. , Yates, J. , Papadopoulou, C. , Robbiani, D. , Bieberich, F. , Vazquez‐Lombardi, R. et al., Platypus: an open‐access software for integrating lymphocyte single‐cell immune repertoires with transcriptomes. 2020. 14: lqab023. 10.1093/nargab/lqab023 PMC8046018 33884369

[37] Csardi, G. and Nepusz, T. , The igraph software package for complex network research. Int. J. Compl. Syst. 2006. 1695: 1–9.

[38] Robinson, M. D. , McCarthy, D. J. and Smyth, G. K. , edgeR: a Bioconductor package for differential expression analysis of digital gene expression data. Bioinformatics 2010. 26: 139–140. 19910308 10.1093/bioinformatics/btp616 PMC2796818

[39] Hastie, K. M. , Zandonatti, M. A. , Kleinfelter, L. M. , Heinrich, M. L. , Rowland, M. M. , Chandran, K. , Branco, L. M. et al., Structural basis for antibody‐mediated neutralization of Lassa virus. Science 2017. 356: 923–928. 28572385 10.1126/science.aam7260 PMC6007842

[40] Mason, D. M. , Friedensohn, S. , Weber, C. R. , Jordi, C. , Wagner, B. , Meng, S. and Reddy, S. T. , Deep learning enables therapeutic antibody optimization in mammalian cells. 2019. 10.1101/617860

[41] Butler, A. , Hoffman, P. , Smibert, P. , Papalexi, E. and Satija, R. , Integrating single‐cell transcriptomic data across different conditions, technologies, and species. Nat. Biotechnol. 2018. 36: 411–420. 29608179 10.1038/nbt.4096 PMC6700744

[42] Wickham, H. and Wickham, M. H. , The ggplot package. 2007.

[43] Wagih, O. , ggseqlogo: a versatile R package for drawing sequence logos. Bioinformatics 2017. 33: 3645–3647. 29036507 10.1093/bioinformatics/btx469

[44] Blanc, P. , Moro‐Sibilot, L. , Barthly, L. , Jagot, F. , This, S. , de Bernard, S. and Buffat, L. et al., Mature IgM‐expressing plasma cells sense antigen and develop competence for cytokine production upon antigenic challenge. Nat. Commun. 2016. 7: 13600. 27924814 10.1038/ncomms13600 PMC5150646

[45] Pinto, D. , Montani, E. , Bolli, M. , Garavaglia, G. , Sallusto, F. , Lanzavecchia, A. and Jarrossay, D. , A functional BCR in human IgA and IgM plasma cells. Blood 2013. 121: 4110–4114. 23550036 10.1182/blood-2012-09-459289

