# Supplementary material for: Profiling the specificity of clonally expanded plasma cells during chronic viral infection by single‐cell analysis
Source: Eur J Immunol. 2021 Nov 23;52(2):297–311. doi: 10.1002/eji.202149331 (PMC9299196; doi:10.1002/eji.202149331)
Supplement: Supplementary file 1 — Supporting Information [file EJI-52-297-s010.pdf]

Supporting information

Profiling the specificity of clonally expanded plasma cells during chronic viral infection by single-cell analysis

Daniel Neumeier[1], Alessandro Pedrioli[2] , Alessandro Genovese[2], Ioana Sandu[2], Roy Ehling[1], Kai-Lin Hong[1], Chrysa Papadopoulou[1], Andreas Agrafiotis[1,2], Raphael Kuhn[1], Danielle Shlesinger[2], Damiano Robbiani[1], Jiami Han[1], Laura Hauri[1], Lucia Csepregi[1], Victor Greiff[3], Doron Merkler[4,5], Sai T. Reddy[1,*], Annette Oxenius[2,*], Alexander Yermanos[1,2,4,*]

[1]Department of Biosystems Science and Engineering, ETH Zurich, Basel, Switzerland
[2]Institute of Microbiology, ETH Zurich, Zurich, Switzerland
[3]Department of Immunology, University of Oslo, Oslo, Norway
[4]Department of Pathology and Immunology, University of Geneva, Geneva, Switzerland
[5]Division of Clinical Pathology, Geneva University Hospital, Geneva, Switzerland
*Correspondence: ayermanos@gmail.com ; aoxenius@micro.biol.ethz.ch ; sai.reddy@bsse.ethz.ch

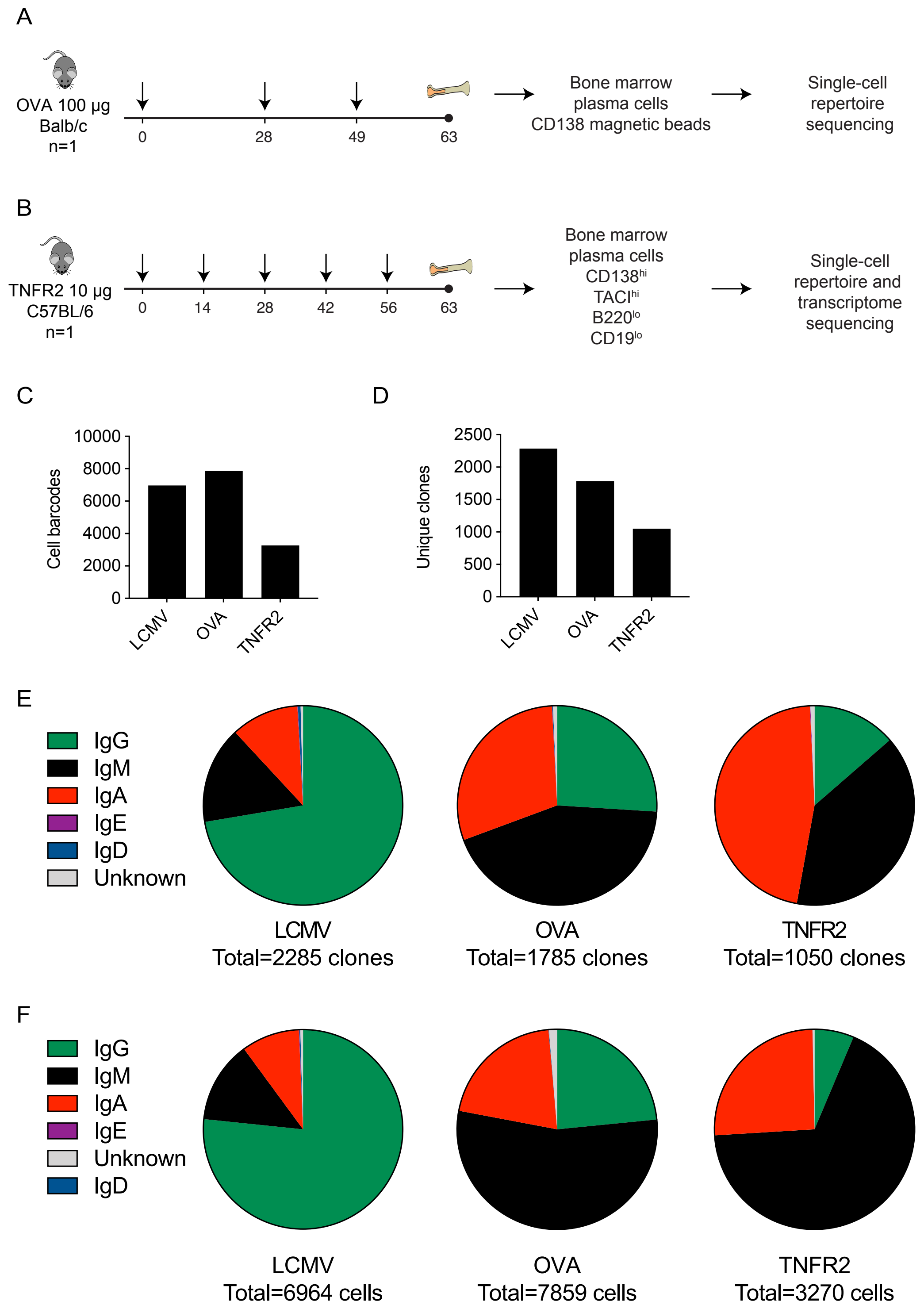

Figure S1. Experimental overview of immunization schemes and antibody repertoire statistics.
A. Serial immunization scheme with ovalbumin (OVA) and subsequent bone marrow plasma cell (BM PC) isolation using CD138+ magnetic beads. Subsequent antibody repertoire libraries were prepared using the 5' single-cell immune profiling kit from 10X Genomics. B. Serial immunization scheme with the extracellular region of human tumor necrosis factor receptor 2 (TNFR2) and subsequent bone marrow plasma cell (BM PC) isolation using flow cytometry. BM PCs were sorted based on $CD138^{hi}$, $TACI^{hi}$,

B220^{lo}, CD19^{lo} . Subsequent antibody repertoire and transcriptome libraries were prepared using the 5' single-cell immune profiling kit from 10X Genomics. C. The total number of cells recovered in each infected or immunized mouse. Only cells containing exactly one variable heavy (V_H) and variable light (V_L) chain were considered. D. The total number of clones recovered in each infected or immunized mouse. Clone was determined by grouping those B cells containing identical CDRH3+CDRL3 amino acid sequences. Only cells containing exactly one variable heavy (V_H) and variable light (V_L) chain were considered. E-F. Fraction of clones (E) and cells (F) corresponding to a given isotype in each infected or immunized mouse. Clone was determined by grouping those B cells containing identical CDRH3+CDRL3 amino acid sequences. Only cells containing exactly one variable heavy (V_H) and variable light (V_L) chain were considered.

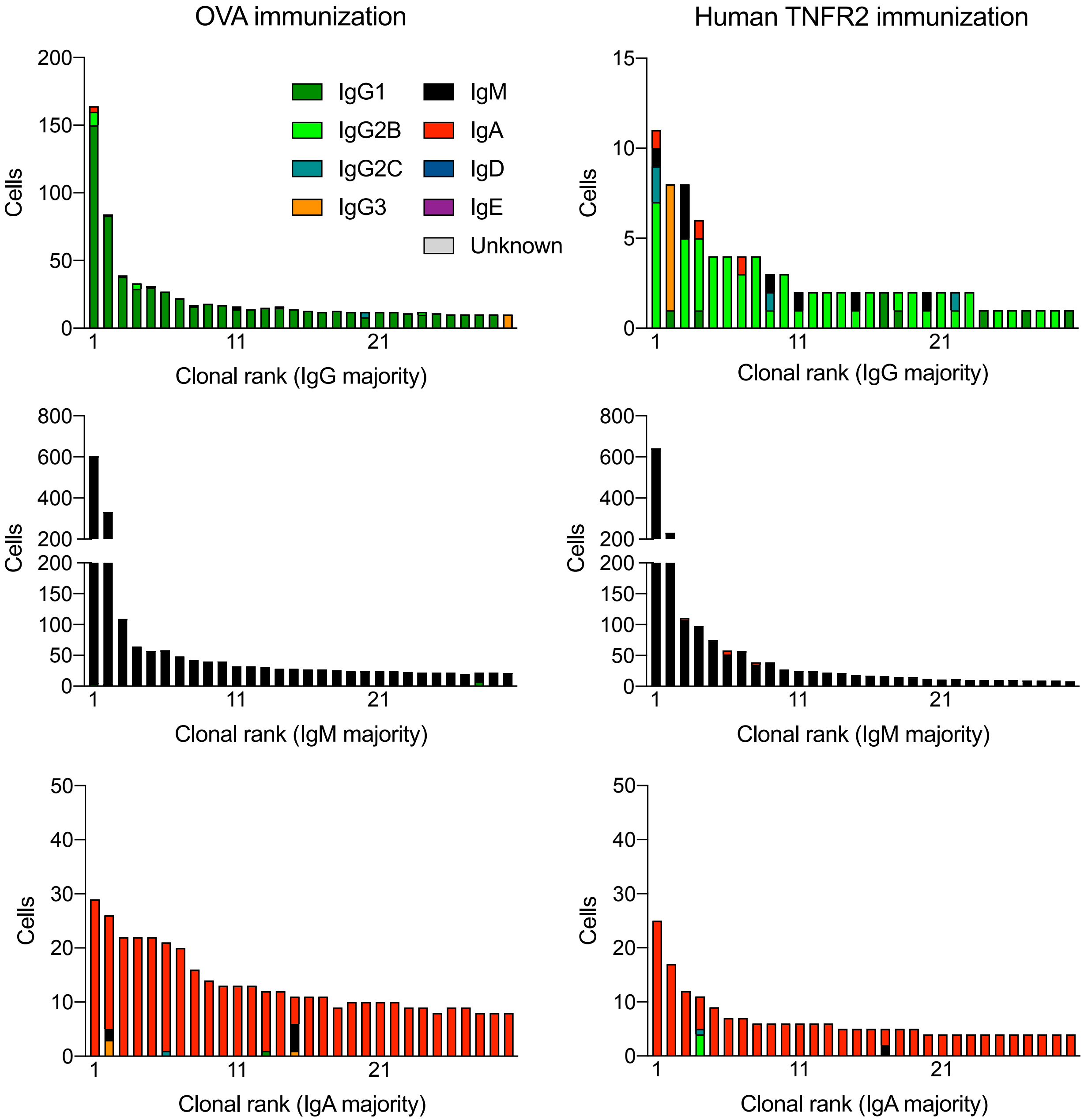

Figure S2. Clonal expansion of bone marrow plasma cells (BM PC) separated by isotype for OVA and TNFR2 immunized mice. The number of distinct cell barcodes belonging to the top 30 clones is visualized. Clone was determined by grouping those B cells containing identical CDRH3+CDRL3 amino acid sequences. Only cells containing exactly one variable heavy (V_H) and variable light (V_L) chain were considered. Color corresponds to isotype per each cell.

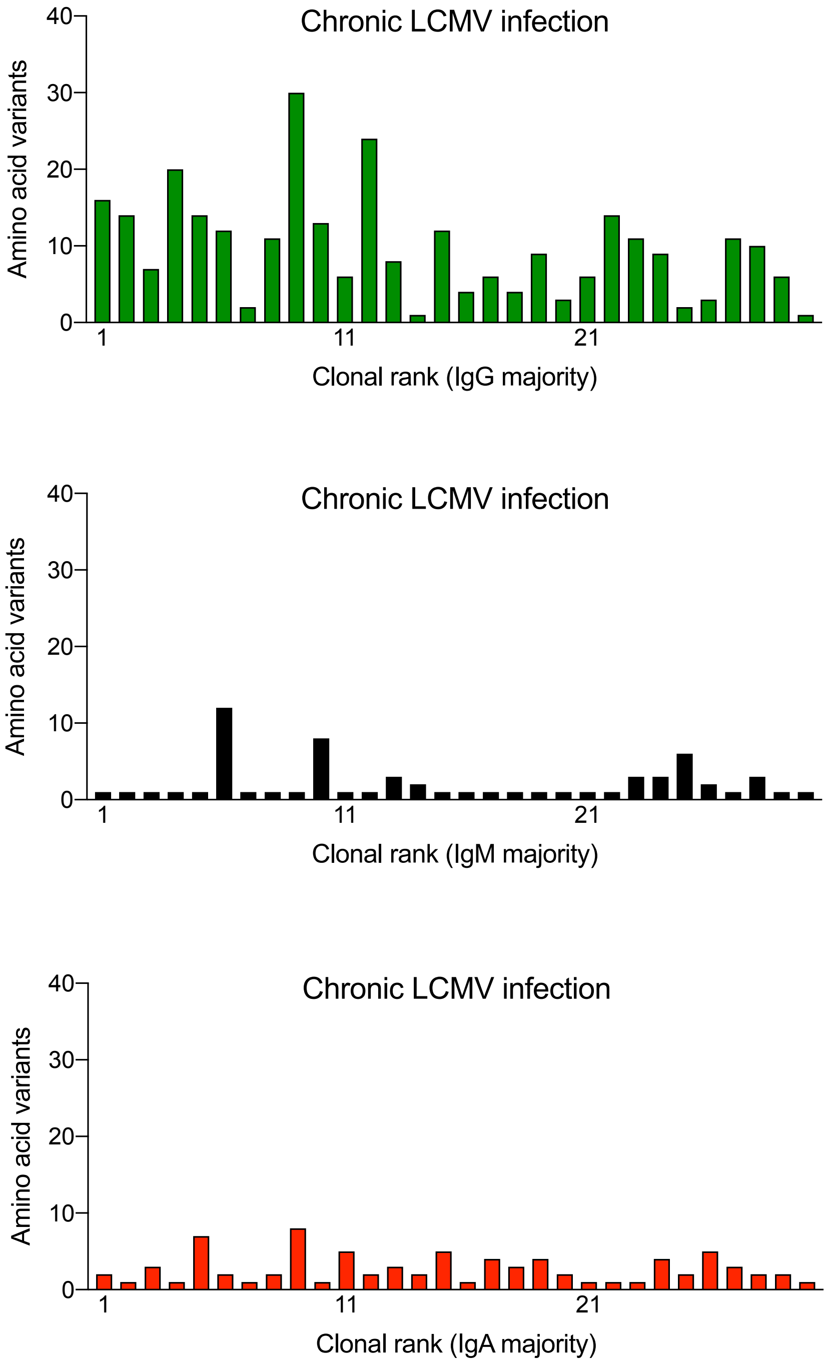

Figure S3. Number of full-length (paired V_H+V_L) amino acid variants for the 30 most expanded clones separated by isotype in the bone marrow plasma cell repertoire following chronic LCMV infection. Clone was determined by grouping those B cells containing identical CDRH3+CDRL3 amino acid sequences.

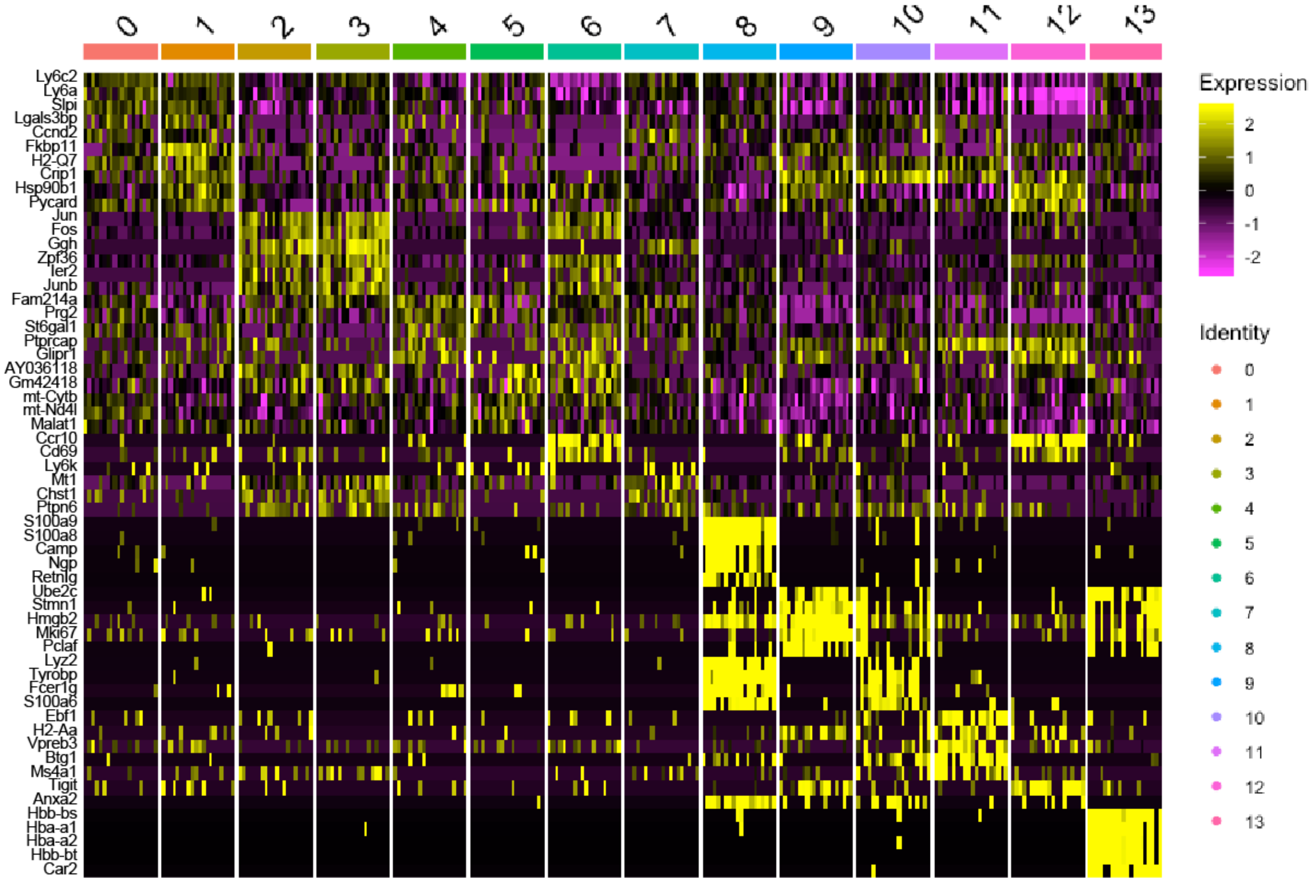

Figure S4. Top differentially expressed genes for each transcriptional cluster of the bone marrow plasma cells from mice either chronically infected with LCMV or immunized with TNFR2. Heatmap intensity corresponds to normalized expression. Each column represents a single cell and each row corresponds to a single gene. The top five genes based on average log fold change (logFC) have been selected for each cluster. All displayed genes had an adjusted p value less than or equal to 0.01.

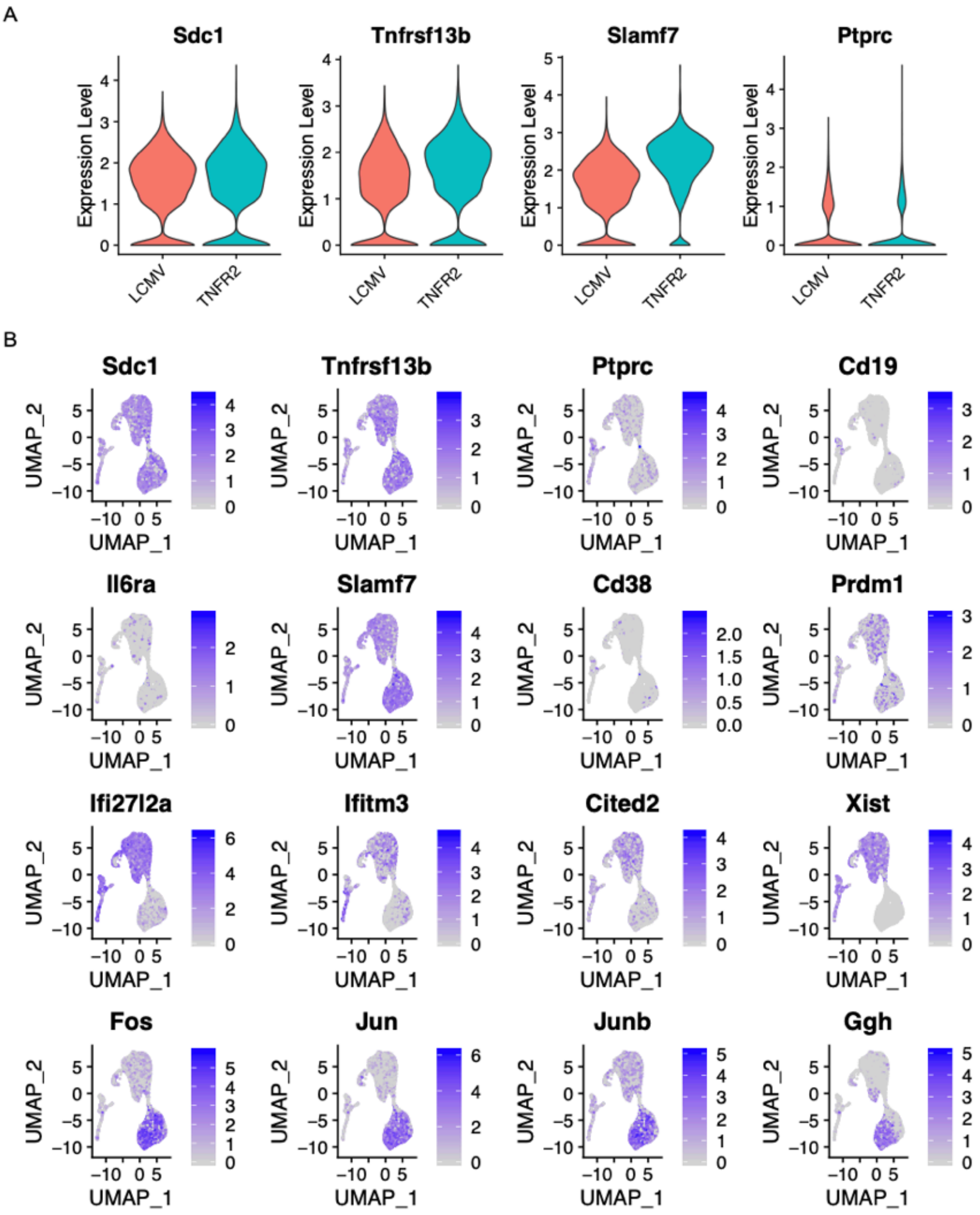

Figure S5. Differentially expressed genes between the bone marrow plasma cells from mice either chronically infected with LCMV or immunized with human TNFR2. (A) Normalized expression of plasma cell genes *Sdc1* (*CD138*), *Tnfrsf13b* (*TACI*), *Slamf7*, and *Ptprc* (*B220*) for either mice infected with LCMV or immunized with human TNFR2. (B) Uniform manifold approximation project (UMAP) plots showing normalized gene expression for selected genes.

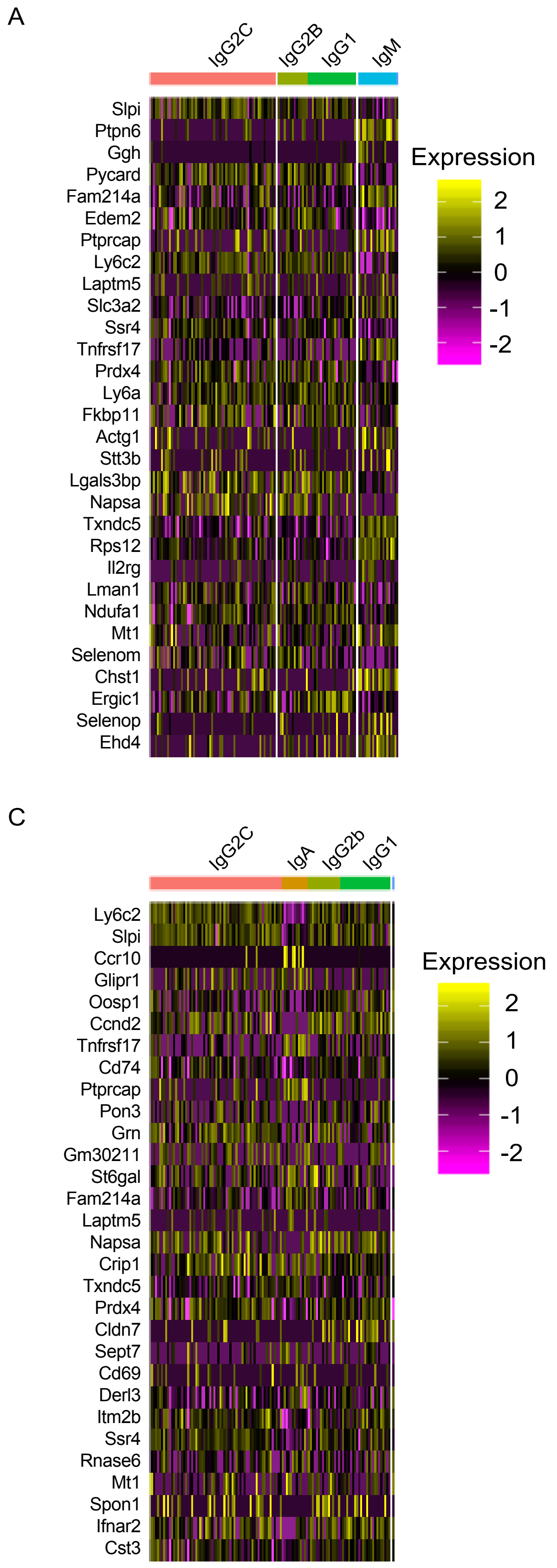
A
IgG2C
IgG2B
IgG1
IgM
Slpi
Ptpn6
Ggh
Pycard
Fam214a
Edem2
Ptprcap
Ly6c2
Laptm5
Slc3a2
Ssr4
Tnfrsf17
Prdx4
Ly6a
Fkbp11
Actg1
Stt3b
Lgals3bp
Napsa
Txndc5
Rps12
Il2rg
Lman1
Ndufa1
Mt1
Selenom
Chst1
Ergic1
Selenop
Ehd4
Expression
2
1
0
-1
-2
B
IgA
IgM
Glipr1
Ccr10
Mt1
Ggh
Cd52
Chst1
Pycard
Ctss
Crip1
Ccnd2
Bst2
Lman1
Ly6c2
Ptpn6
Slamf9
Oosp1
Slc3a2
Edem2
Aa
Trp53inp1
Wnt10a
Rasgrp2
Selenom
Jpt1
St6gal1
Cd74
Srgn
Rasgrp3
Rgcc
Itgb7
Expression
2
1
0
-1
-2
C
IgG2C
IgA
IgG2b
IgG1
Ly6c2
Slpi
Ccr10
Glipr1
Oosp1
Ccnd2
Tnfrsf17
Cd74
Ptprcap
Pon3
Grn
Gm30211
St6gal
Fam214a
Laptm5
Napsa
Crip1
Txndc5
Prdx4
Cldn7
Sept7
Cd69
Derl3
Itm2b
Ssr4
Rnase6
Mt1
Spon1
Ifnar2
Cst3
Expression
2
1
0
-1
-2

Figure S6. Differentially expressed genes between cells expressing different isotypes. (A) Differentially expressed genes between IgG vs IgM of plasma cells coming from an LCMV infected mouse. (B) Differentially expressed genes between IgM vs IgA of plasma cells coming from an LCMV infected mouse. (C) Differentially expressed genes between IgG vs IgA of plasma cells coming from an LCMV infected mouse. Heatmap intensity corresponds to normalized expression. Each column represents a single cell and each row corresponds to a single gene. The top 30 genes based on average log fold change (logFC) for each isotype comparison were selected. All displayed genes had an adjusted p value less than or equal to 0.01.

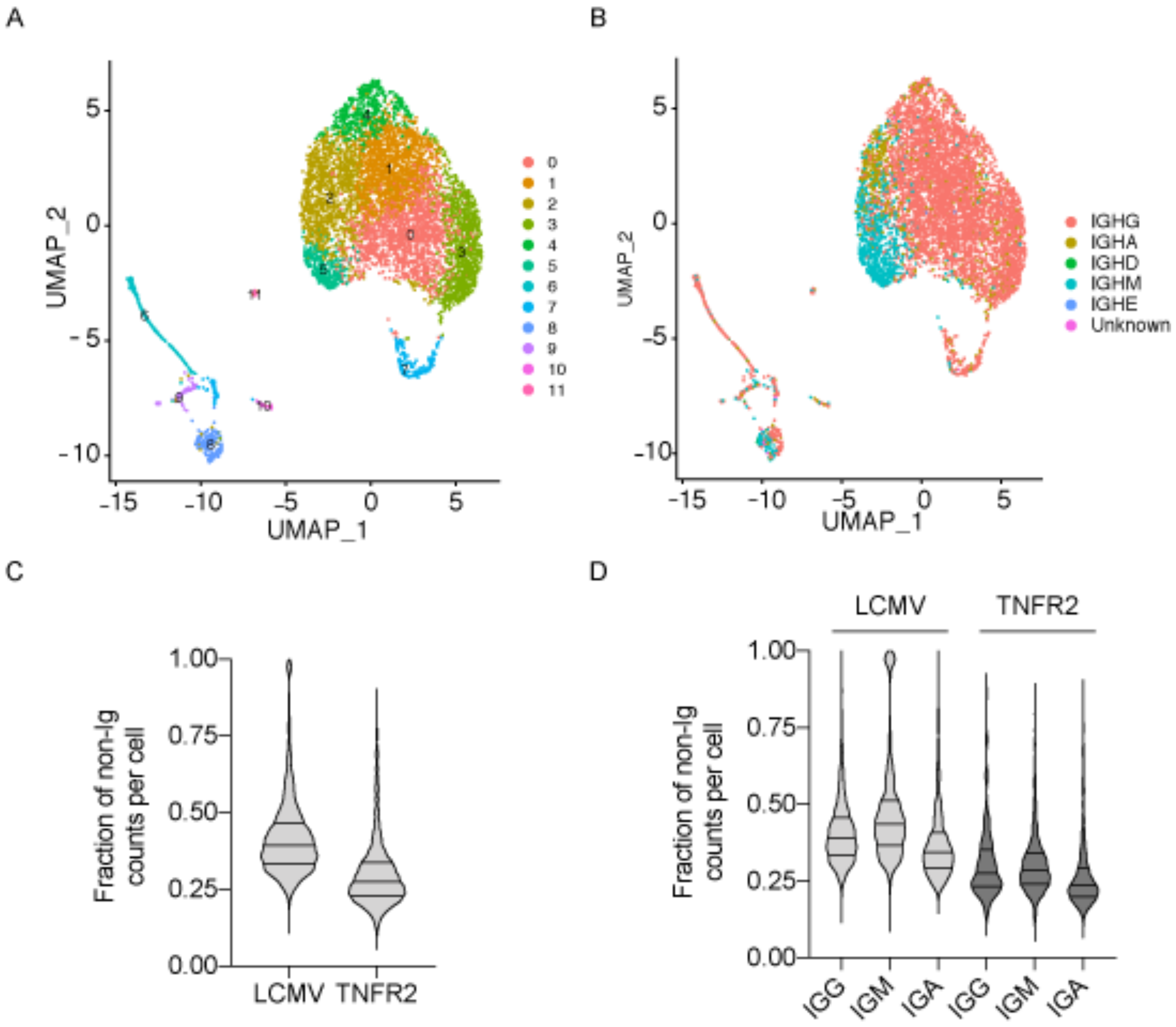

Figure S7. A-B. Uniform manifold approximation projection (UMAP) based total gene expression for bone marrow plasma cell (BM PC) repertoire following LCMV infection colored by either transcriptional cluster (A) or isotype (B). (C-D) Fraction of total gene counts that did not correspond to the adaptive immune receptor genes following either LCMV infection or TNFR2 immunization.

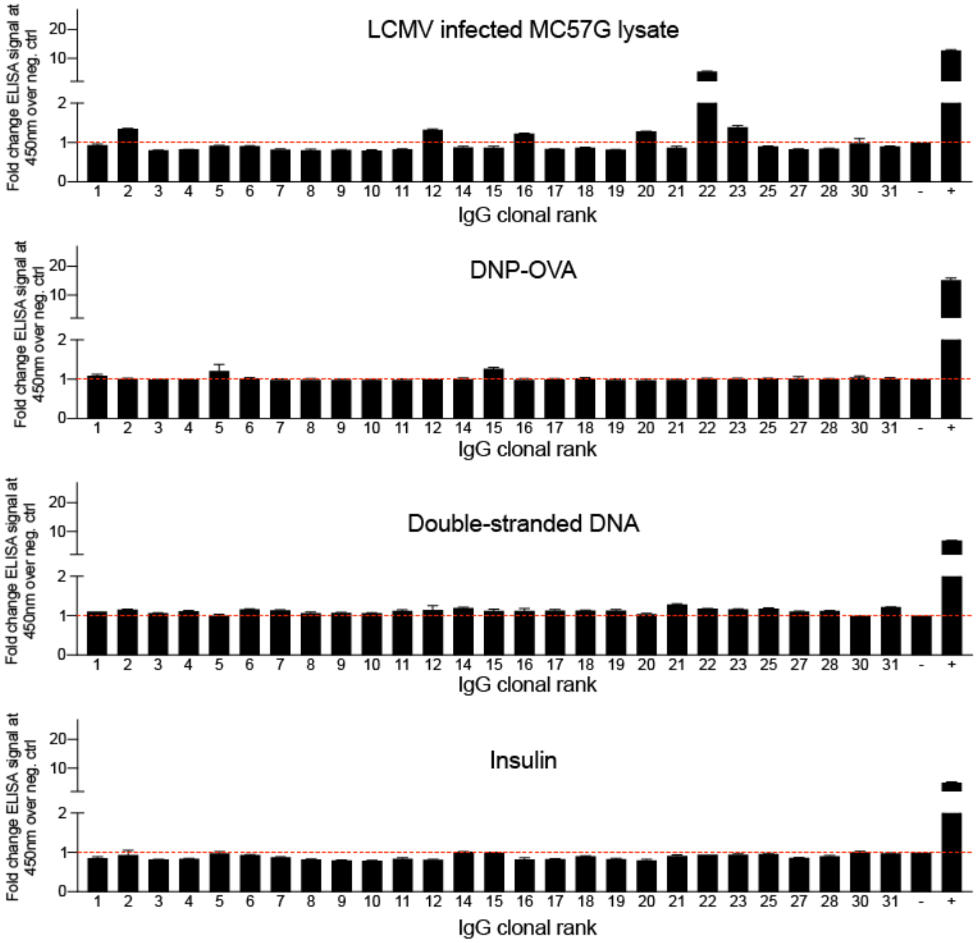

Figure S8. Clonally expanded plasma cells produce virus-specific and potentially autoreactive antibodies following chronic viral infection. The ELISA signal of duplicate ELISA measurements at 450 nm is shown relative to a negative background control (red dotted line indicates background level). Control antibodies used are listed in the methods section.

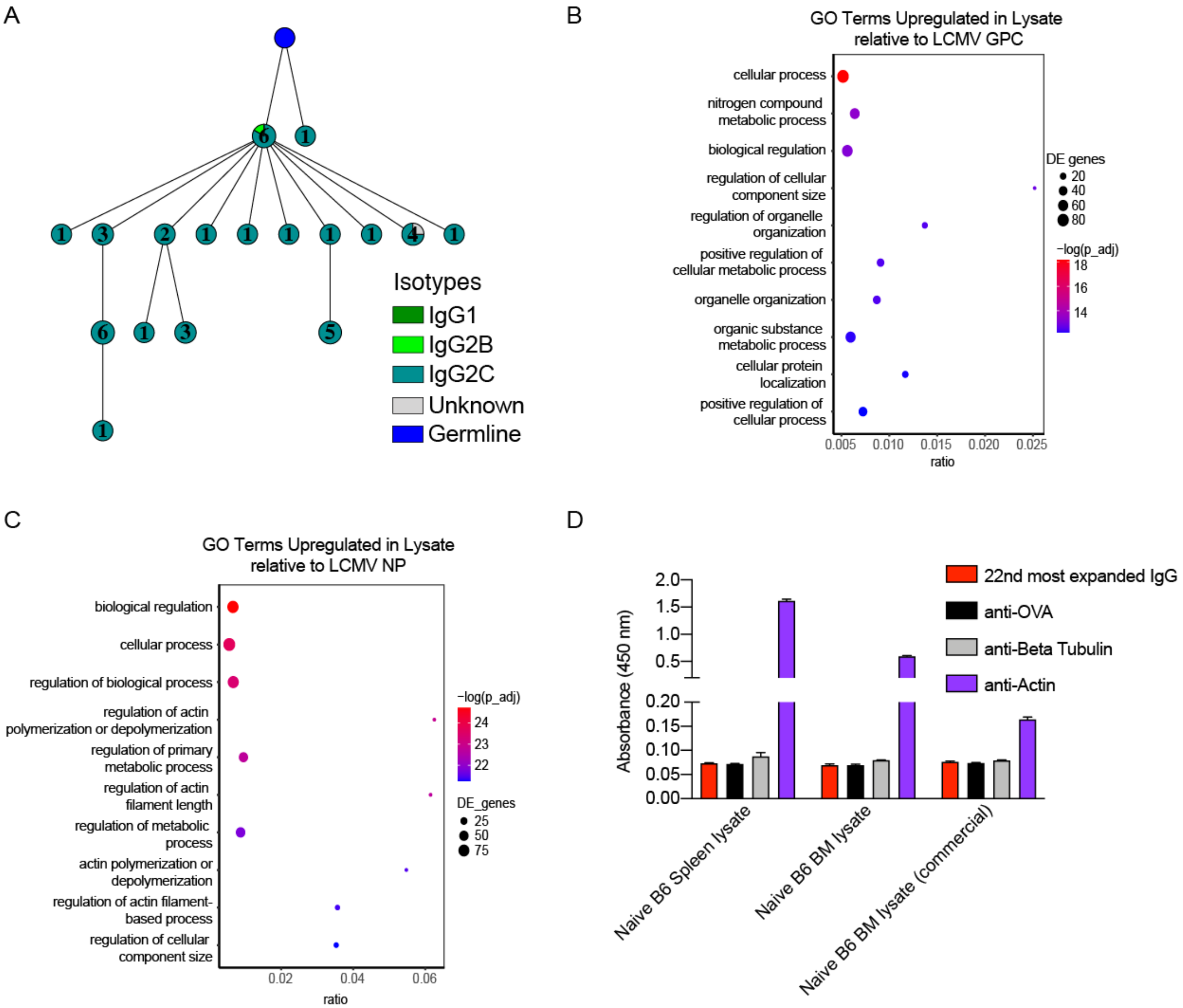

Figure S9. Features of the lysate-specific clone. A. Mutational network of the IgG clone binding MC57G lysate. Nodes represent unique antibody variants (combined V_H+V_L nucleotide sequence) and edges demonstrate sequences with the smallest separation calculated by edit distance. Node color corresponds to transcriptional cluster from 3A. The size and label of the nodes indicate how many cells express each full-length antibody variant. Clone was determined by grouping those B cells containing identical CDRH3+CDRL3 amino acid sequences. Only cells containing exactly one variable heavy (V_H) and variable light (V_L) chain were considered. The isotype was determined as the isotype corresponding to the majority of cells within one clone. The germline node represents the unmutated reference sequence determined by 10x Genomics cellranger. B-C. Gene ontology (GO) term enrichment of upregulated genes in MC57G lysate binder compared to either LCMV GP or LCMV NP binders. The color of each dot corresponds to adjusted p value. The size of the dot corresponds to the number of genes. Ratio corresponds to the number of differentially genes relative to the number of total genes corresponding to each GO term. D. Repetition of ELISA on lysate from various tissues of naive C57BL/6 mice. Error bars indicate the standard error of mean.

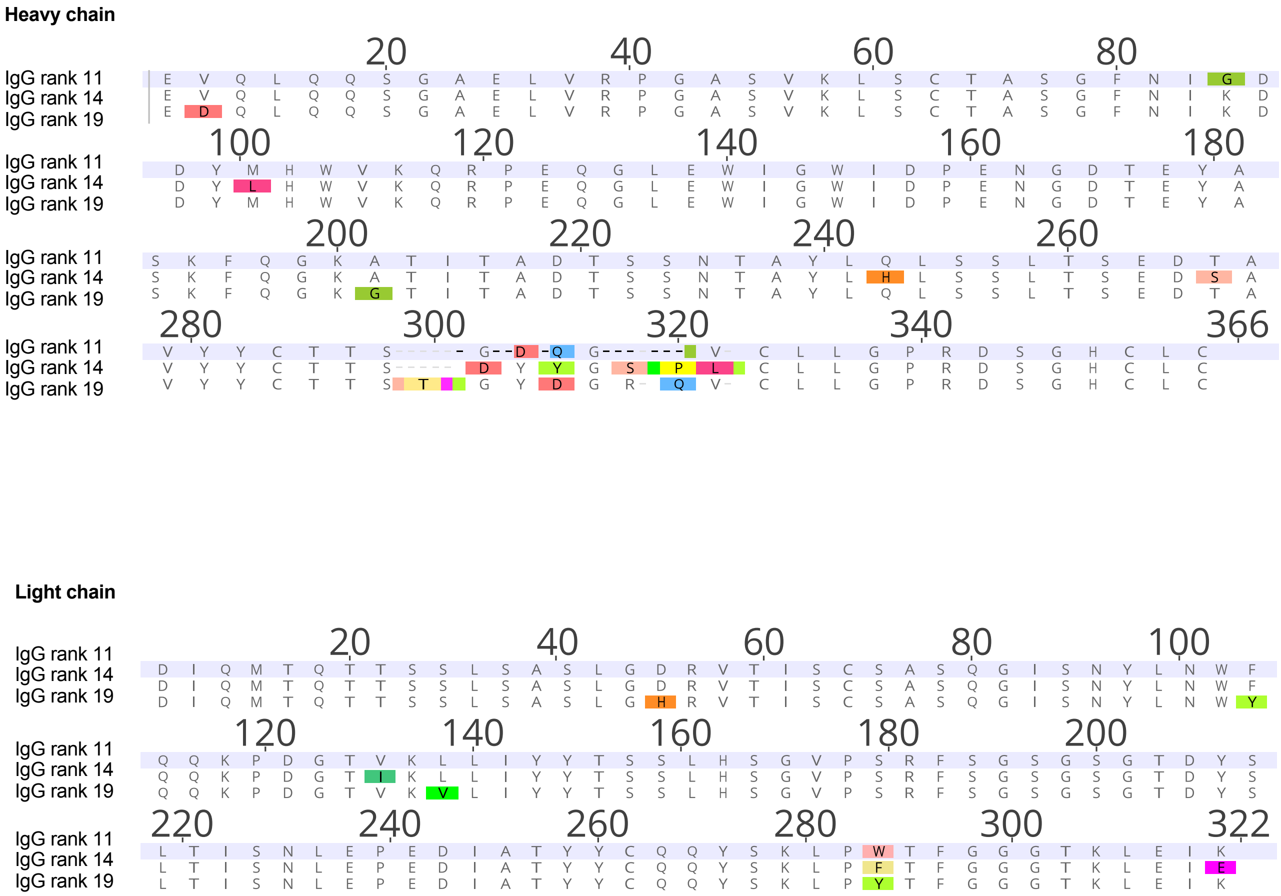

Figure S10. V_H and V_L amino acid alignment of the three LCMV GPC specific clones.

A

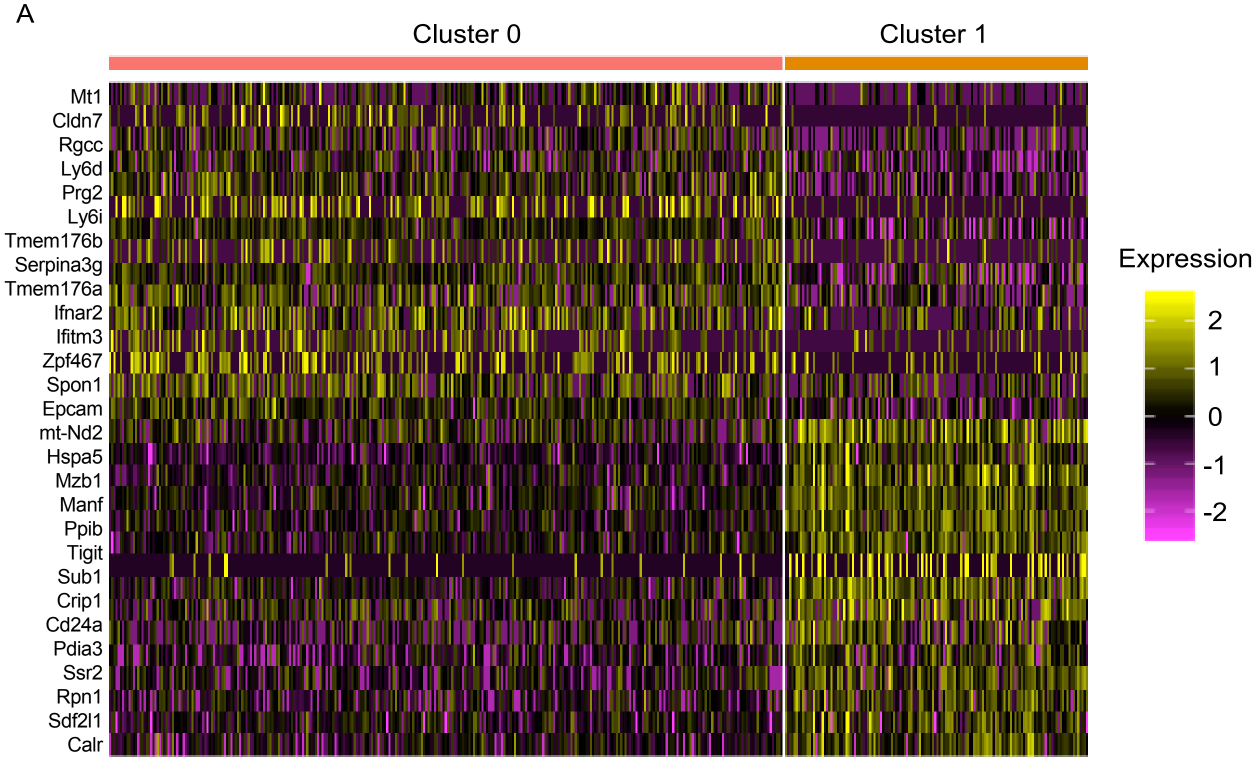
Cluster 0
Cluster 1
Mt1
Cldn7
Rgcc
Ly6d
Prg2
Ly6i
Tmem176b
Serpina3g
Tmem176a
Ifnar2
Ifitm3
Zpf467
Spon1
Epcam
mt-Nd2
Hspa5
Mzb1
Manf
Ppib
Tigit
Sub1
Crip1
Cd24a
Pdia3
Ssr2
Rpn1
Sdf2l1
Calr
Expression
2
1
0
-1
-2

B

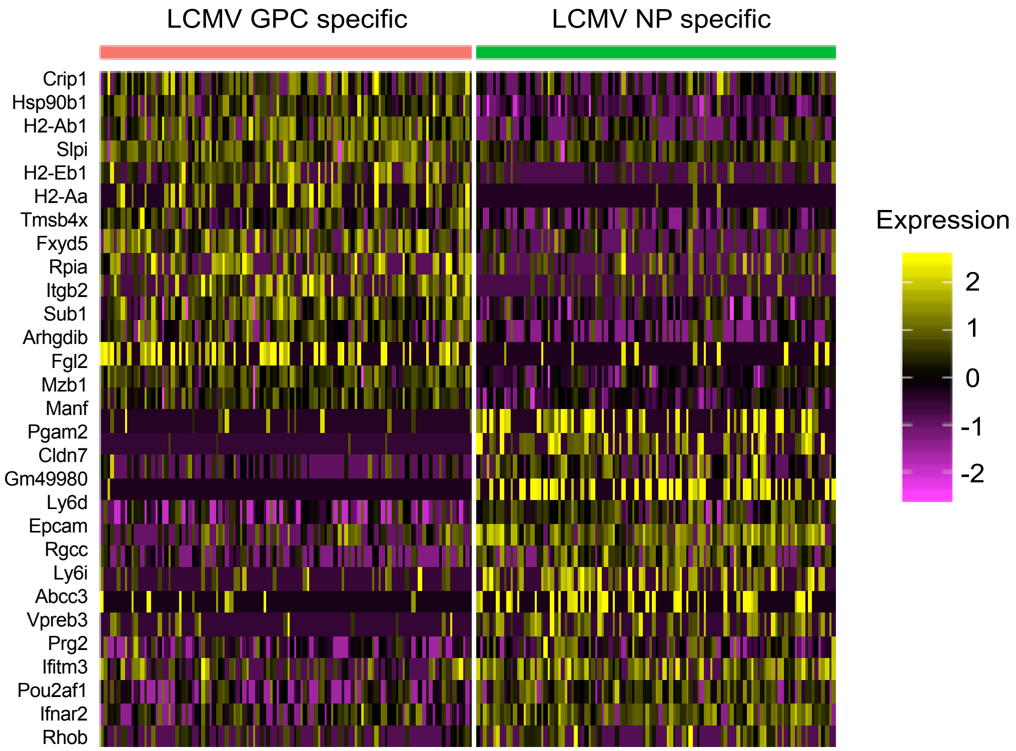
LCMV GPC specific
LCMV NP specific
Crip1
Hsp90b1
H2-Ab1
Slpi
H2-Eb1
H2-Aa
Tmsb4x
Fxyd5
Rpia
Itgb2
Sub1
Arhgdib
Fgl2
Mzb1
Manf
Pgam2
Cldn7
Gm49980
Ly6d
Epcam
Rgcc
Ly6i
Abcc3
Vpreb3
Prg2
Ifitm3
Pou2af1
Ifnar2
Rhob
Expression
2
1
0
-1
-2

Figure S11. Signature genes expressed by plasma cells producing either NP or GPC specific antibodies. A. Differentially expressed genes between cells located in either cluster 0 or cluster 1. Heatmap intensity corresponds to normalized expression. Each column represents a single cell and each row corresponds to a single gene. The top 40 differentially expressed genes based on average log fold change (logFC) were selected. All displayed genes had an adjusted p value less than or equal to 0.01. B. Differentially expressed genes between the plasma cells producing either NP or GPC specific antibodies. Each column represents a single cell and each row corresponds to a single gene. The top 40 differentially expressed genes based on average logFC were selected. All displayed genes had an adjusted p value less than or equal to 0.01.

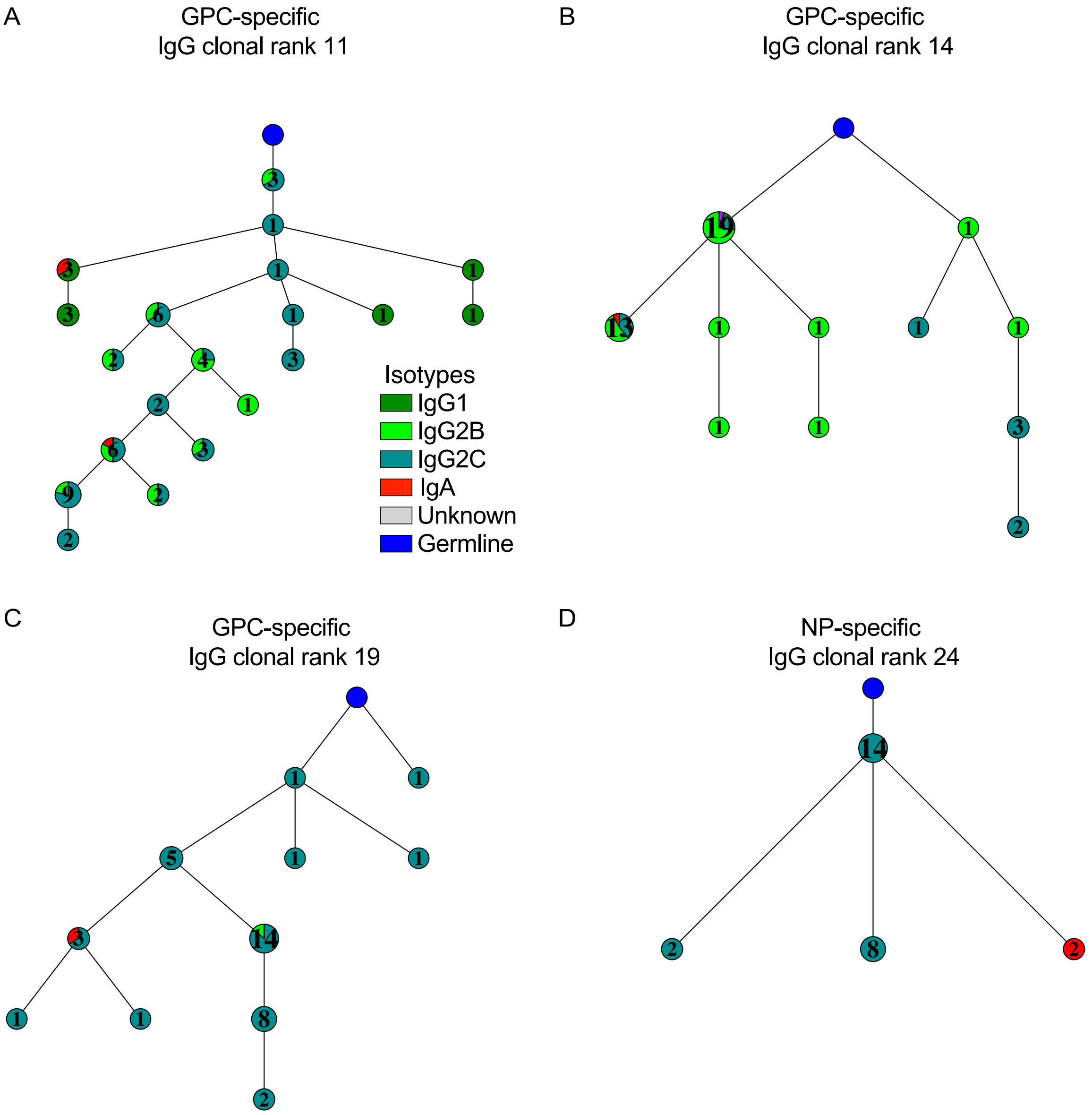

Figure S12. Virus-specific mutational networks displaying isotype distribution. Nodes represent unique antibody variants (combined V_H+V_L nucleotide sequence) and edges demonstrate sequences with the smallest separation calculated by edit distance. Node color corresponds to isotype distribution for each cell. The size and label of the nodes indicate how many cells express each full-length antibody variant. Clone was determined by grouping those B cells containing identical CDRH3+CDRL3 amino acid sequences. Only cells containing exactly one variable heavy (V_H) and variable light (V_L) chain were considered. The germline node represents the unmutated reference sequence determined by 10x Genomics cellranger.

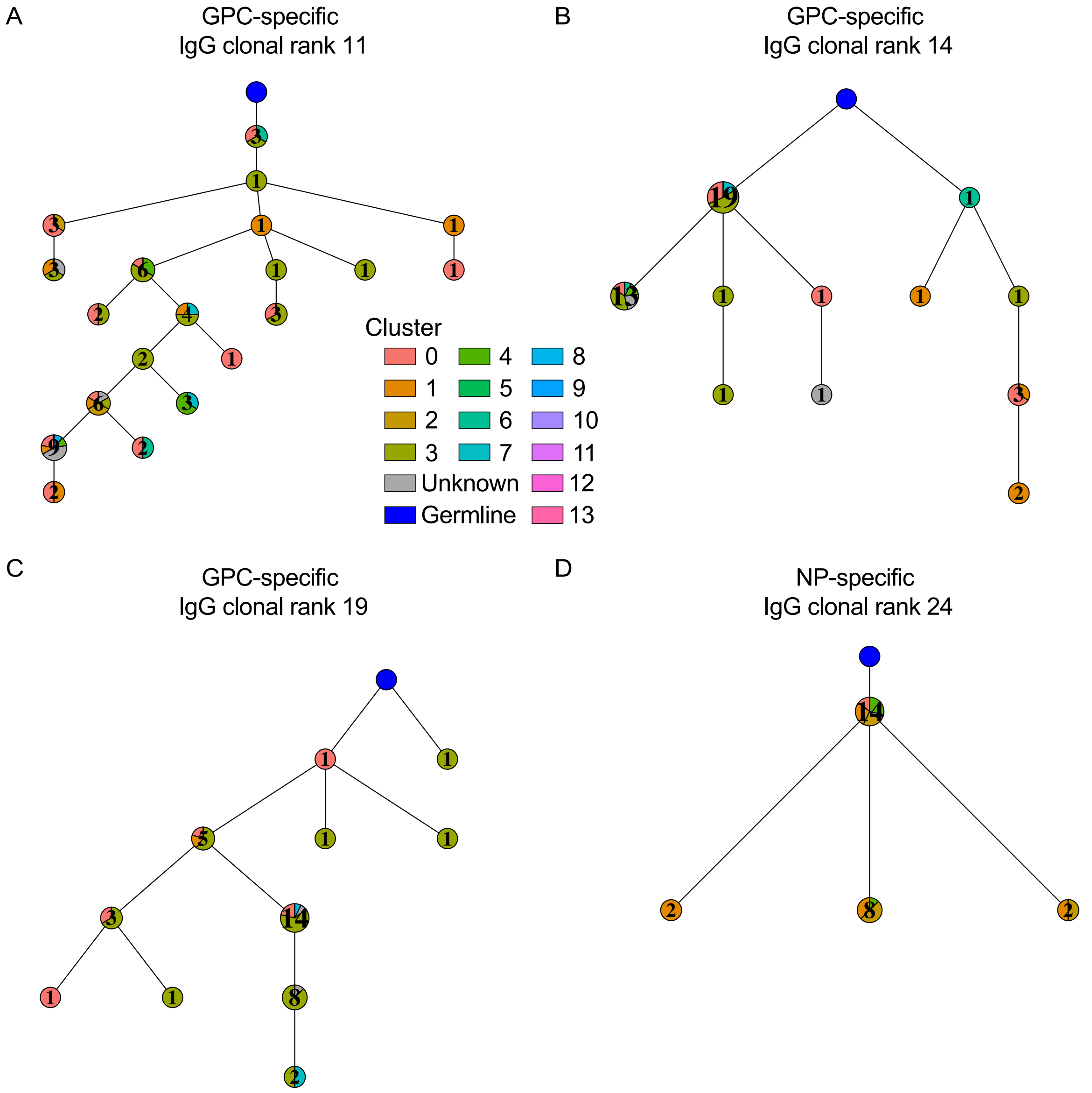

Figure S13. Virus-specific mutational networks displaying transcriptional cluster distribution. Nodes represent unique antibody variants (combined V_H+V_L nucleotide sequence) and edges demonstrate sequences with the smallest separation calculated by edit distance. Node color corresponds to transcriptional cluster distribution for each cell. The size and label of the nodes indicate how many cells express each full-length antibody variant. Clone was determined by grouping those B cells containing identical CDRH3+CDRL3 amino acid sequences. Only cells containing exactly one variable heavy (V_H) and variable light (V_L) chain were considered. The germline node represents the unmutated reference sequence determined by 10X Genomics cellranger.

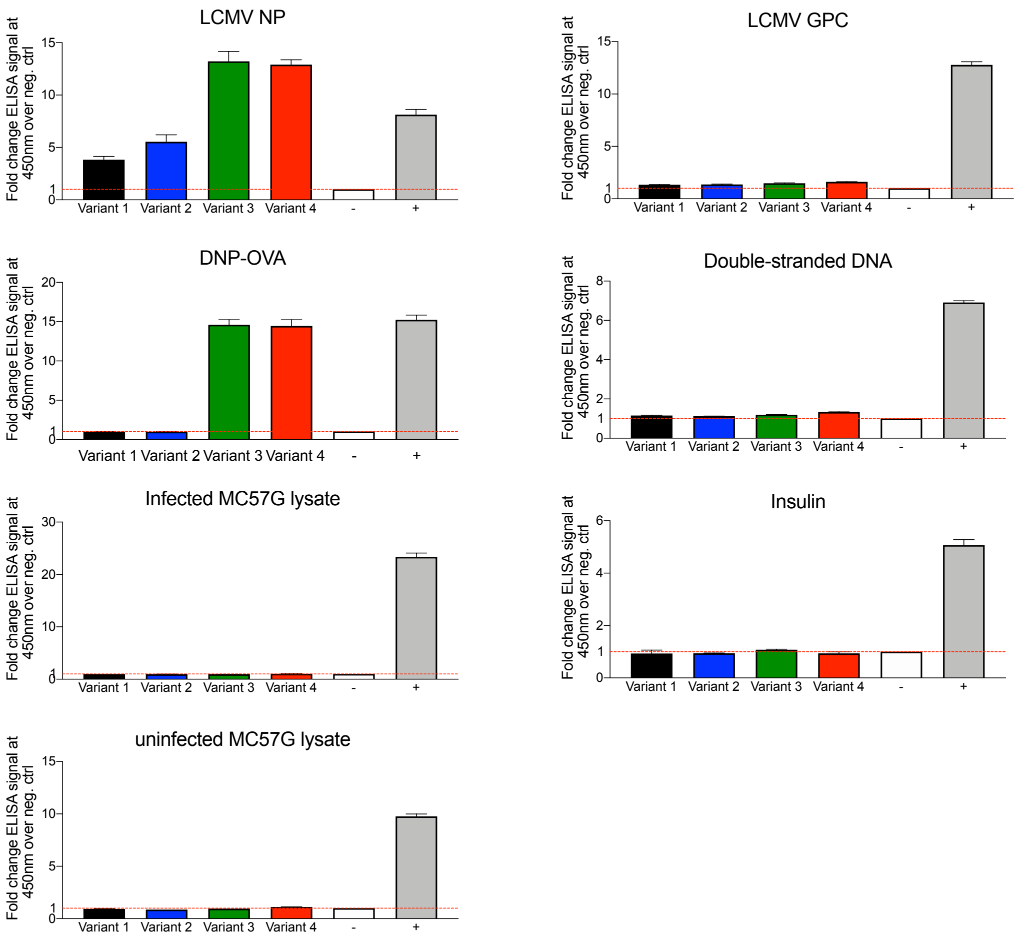

Figure S14. DNP-OVA cross-reactive antibody variants do not react with other antigens and cell lysates. The ELISA signal of duplicate ELISA measurements at 450 nm is shown relative to a negative background control (red dotted line indicates background level). Control antibodies used are listed in the methods section.

Supplementary table S1. Top 200 cluster defining genes.
Supplementary table S2. LCMV vs TNFR2 Differentially expressed genes
Supplementary table S3. Differentially expressed genes between IgG and IgM following LCMV infection
Supplementary table S4. Differentially expressed genes between IgA and IgM following LCMV infection
Supplementary table S5. Differentially expressed genes between IgG and IgA following LCMV infection
Supplementary table S6. Validated antibodies following LCMV infection
Supplementary table S7. Differentially expressed genes between lysate- and GPC-specific PCs following LCMV infection
Supplementary table S8. Differentially expressed genes between lysate- and NP-specific PCs following LCMV infection
Supplementary table S9. Differentially expressed genes between cluster 0 and cluster 1.
Supplementary table S10. Differentially expressed genes between GPC- and NP-specific PCs following LCMV infection
